# A county-level indicator framework for assessing schistosomiasis transmission risk in post-transmission-interruption China

**DOI:** 10.1186/s40249-026-01453-6

**Published:** 2026-05-18

**Authors:** Andong Xu, Hong Zhu, Jing Xu, Xiaowei Shan, Yi Dong, Hongqiong Wang, Chao Lv, Shiyi Huo, Shizhu Li

**Affiliations:** 1 National Institute of Parasitic Diseases, Chinese Center for Disease Control and Prevention (Chinese Center for Tropical Diseases Research), National Health Commission Key Laboratory of Parasite and Vector Biology, WHO Collaborating Centre for Tropical Diseases, National Center for International Research on Tropical Diseases, Ministry of Science and Technology, Shanghai, 200025 China; 2 Hubei Center for Disease Control and Prevention, Hubei Provincial Academy of Preventive Medicine, Wuhan, 430079 China; 3 Department of Schistosomiasis Control and Prevention, Yunnan Institute of Endemic Disease Control and Prevention, Kunming, 650500 China; 4 Yunnan Key Laboratory of Natural Focus Disease Control Technology, Dali, 671000 China; 5 School of Global Health, Shanghai Jiao Tong University School of Medicine and Chinese Center for Tropical Diseases Research, Shanghai, 200025 China

**Keywords:** Schistosomiasis, Transmission interruption, Transmission risk, Indicator framework, Delphi, Entropy weight, Fuzzy membership

## Abstract

**Background:**

In China’s post-transmission-interruption stage of schistosomiasis control, infection signals are often sparse, and conventional surveillance indicators focused on infection detection may not adequately capture residual risk across heterogeneous ecological settings. This study aimed to develop an operational indicator framework for assessing schistosomiasis transmission risk and supporting routine risk assessment and management in this stage.

**Methods:**

A county-level indicator framework for schistosomiasis transmission risk assessment was developed through a two-round Delphi consultation and weighted using a combined Delphi-entropy weight method. Using longitudinal data from six pilot counties during 2020–2024, an annual composite risk index (*R*) was calculated by weighted linear aggregation and classified into risk levels with trapezoidal fuzzy membership functions. Robustness was evaluated by perturbing the subjective preference coefficient (α) and examining the consistency of county-year rankings across scenarios using Spearman’s rank correlation.

**Results:**

The final framework comprised three first-level, twelve second-level, and thirty-nine third-level indicators spanning biological, environmental, and social domains. Across the six pilot counties, *R* ranged from 0.18 to 0.44 and the overall risk level was predominantly low. Nonetheless, distinct county-level risk profiles were observed between lake/marshland and mountainous settings. Risk rankings remained highly consistent under α perturbations (*ρ* = 0.909–0.998), indicating good robustness of the assessment results.

**Conclusions:**

This framework translates multidimensional determinants relevant to the re-establishment of schistosomiasis transmission into interpretable county-level risk profiles. It provides an operational tool to support targeted surveillance and more efficient allocation of control resources in low-endemicity contexts.

**Supplementary Information:**

The online version contains supplementary material available at 10.1186/s40249-026-01453-6.

## Background

Schistosomiasis is a zoonotic parasitic disease caused by *Schistosoma* spp. that remains endemic mainly in tropical and subtropical regions [1, 2]. As a major neglected tropical disease, it causes chronic and disabling morbidity and continues to impose substantial health and socioeconomic burdens [3]. In 2021, the World Health Organization (WHO) set targets to eliminate schistosomiasis as a public health problem in all endemic countries by 2030 and to interrupt transmission in a subset of countries [4].

China was once one of the countries most severely affected by *Schistosoma japonicum*, with endemic areas concentrated mainly along the Yangtze River Basin [5]. Owing to marked ecological and environmental heterogeneity, these areas are generally classified into two major types: lake/marshland regions and mountainous regions [6]. To address this heterogeneous transmission ecology and the complex life cycle of *S. japonicum*, China has implemented successive control strategies that have reduced transmission to historically low levels [7, 8]. China has also adopted criteria for transmission interruption that are more stringent than those proposed by WHO, requiring zero infections in humans, livestock, and snails for five consecutive years, followed by a further five-year consolidation period [9]. In 2023, China completed the first five-year period with zero infections detected in humans, livestock, and snails [10].

In infectious disease epidemiology, transmission risk is generally understood as the likelihood that, under specific spatiotemporal conditions, a pathogen is transmitted from an infectious source to susceptible hosts and gives rise to onward spread [11]. As transmission declines to very low levels, however, residual risk becomes increasingly difficult to detect. For schistosomiasis, this risk arises from the possibility that cercariae may still infect humans or animals through contact with infested water and that transmission may subsequently be sustained or re-established through interactions among infection sources, intermediate hosts, and local ecological and behavioral conditions [12, 13]. Current studies suggest that persistent snail habitats [14], multiple potential host species [15], and environmental, behavioral, and health-system factors [16] acting together may all contribute to such latent risk.

Several approaches, including transmission dynamics modelling, spatiotemporal analysis, and machine learning, have been used to assess schistosomiasis risk [17–21]. However, in settings approaching elimination, where infections in humans and livestock are rarely detected and surveillance data are dominated by zeros, these methods may face challenges in model calibration and predictive stability. As a result, environmental indicators such as snail distribution are often used as proxies for risk [22, 23]. Although useful, such proxies alone may not adequately capture the broader determinants of residual transmission risk after transmission interruption.

An effective risk assessment framework for this stage should therefore reflect the main components of schistosomiasis transmission, including potential infection sources, snail-related ecological conditions, environmental suitability, opportunities for human and animal water contact, and the capacity of surveillance and control systems to detect and respond to residual risk. Indicator-based approaches may offer a practical alternative because they can integrate heterogeneous, multisource information into interpretable risk profiles [24]. However, most existing indicator frameworks have been developed for a single ecological setting, and evidence on their field applicability remains limited [25–27].

In this study, we developed an indicator-based framework to assess the transmission risk of schistosomiasis in China after transmission interruption and applied it in county-level endemic areas in both lake/marshland regions and mountainous regions. This framework may support a more structured assessment of multidimensional residual risk, as well as targeted surveillance and more efficient risk management in settings approaching elimination.

## Methods

### Preparation for the Delphi consultation

#### Literature search strategies

The questionnaire was developed on the basis of a systematic literature search of studies on schistosomiasis transmission and transmission risk assessment published between 1 January 2015 and 31 December 2024. We searched PubMed, Web of Science, and ScienceDirect, together with major Chinese databases, including China National Knowledge Infrastructure (https://www.cnki.com.cn/), Wanfang (https://www.wanfangdata.com.cn/), and the Chinese Biomedical Literature Database (http://www.sinomed.ac.cn/). The search strategies combined controlled vocabulary and free-text terms, such as: (schistosomiasis) AND (transmission OR risk assessment OR epidemiology OR control measures OR surveillance) AND (*Oncomelania hupensis* OR intermediate host OR animal reservoir); corresponding Chinese search terms were applied in parallel. Relevant policy documents, guidelines, and gray literature that might not be indexed in bibliographic databases were also identified by screening the official websites of the WHO and key Chinese authorities, including the National Health Commission, the Chinese Center for Disease Control and Prevention (CCDC), the Ministry of Water Resources, the Ministry of Agriculture and Rural Affairs, and the National Forestry and Grassland Administration.

**Fig. 1:**
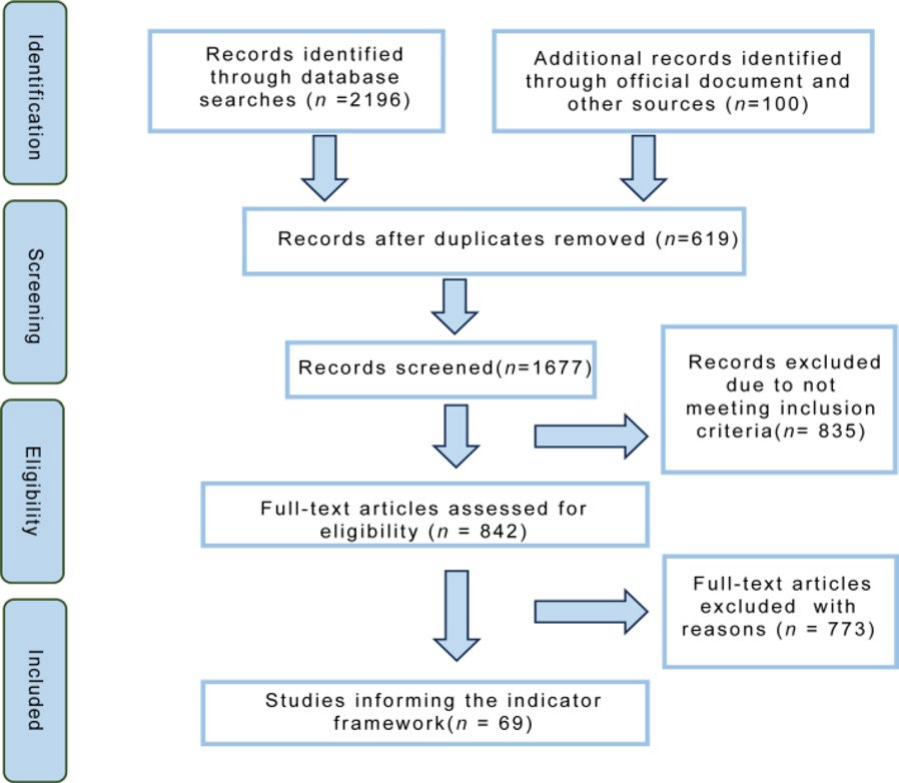
Flow chart of indicator extraction

Studies were included if they addressed schistosomiasis transmission pathways, risk factors, or control interventions and reported qualitative or quantitative indicators that could be directly extracted or derived. Conference abstracts, reviews, case reports, studies focused on molecular epidemiology or laboratory diagnostics, and publications unrelated to schistosomiasis were excluded (Fig. 1).

#### Expert selection

Experts were invited from national-level institutions and the 12 schistosomiasis-endemic provinces in China. The eligibility criteria required experts to hold at least a bachelor’s degree and a professional title of intermediate rank or above. Candidates were also required to have ≥ 10 years of professional experience in schistosomiasis-related control, epidemiological research, or health policy. All participants agreed to complete the full Delphi process.

#### Delphi consultation questionnaire design

The questionnaire consisted of two sections (Additional file 1).

1. Indicator assessment form: Experts rated each indicator on a five-point (1–5) Likert scale across three dimensions: rationality, importance, and operability. Space was provided for qualitative comments and suggestions for revision. The judgement basis (*C*_*a*_) was derived from four sources: practical experience, theoretical evidence, peer awareness, and intuition (Table 1). The familiarity coefficient (*C*_*s*_) reflected each expert’s self-reported familiarity with the indicators (Table 2).
2. Expert characteristics form: Information was collected on sex, age, education, professional title, institutional affiliation, and years of engagement in schistosomiasis-related work.

**Table 1:** Judgment based on the degree of influence

Judgment basis	Degree of influence		
	Large	Medium	Small
Practical experience	0.5	0.4	0.3
Theoretical evidence	0.3	0.2	0.1
Peer awareness	0.1	0.1	0.1
Intuition	0.1	0.1	0.1

**Table 2:** Quantification of self-rated familiarity

Level of familiarity	Quantitative score
Very unfamiliar	0.2
Somewhat unfamiliar	0.4
Neutral	0.6
Familiar	0.8
Extremely familiar	1.0

### Development of the indicator framework

#### Indicator framework construction

The Delphi method is a structured, anonymous expert consultation approach that iteratively refines judgement through multiple rounds of feedback, and is commonly used for indicator selection and subjective weighting [28]. During framework development, candidate indicators were selected primarily with reference to key components of the schistosomiasis transmission chain, including infection sources, snail habitat persistence, environmental suitability, exposure opportunities, and public health response capacity. Based on expert judgement, data availability, the mean scores and coefficients of variation (CVs) for rationality, importance, and operability, and experts’ qualitative comments, indicators were then retained, revised, or deleted.

In round 1: Indicators with a mean score ≥ 3.75 and a CV ≤ 30% were retained. Indicators with a mean score < 3.75 or a CV > 30% were earmarked for revision or deletion. Indicators proposed for addition or modification by at least one third of experts were considered.

In round 2: Indicators with a mean < 3.75 or a CV > 25% were further revised or deleted to yield a stable indicator framework with a high level of expert consensus.

#### Reliability evaluation of the Delphi consultation

The rigor and consistency of the Delphi process were evaluated in terms of three parameters.

1. The positive coefficient was represented by the expert response rate (%).
2. Authority coefficient (*C*_*r*_) was calculated from the judgement basis coefficient (*C*_*a*_) and the familiarity coefficient (*C*_*s*_).
  $${C}_{r}=\frac{({C}_{a}+{C}_{s})}{2}$$
  *C*_*r*_ ≥ 0.70 was considered to indicate high expert authority.
3. Expert consensus: agreement was quantified with Kendall’s coefficient of concordance (*W*), with significance tested with the *χ*^2^ test. Score dispersion was described with the CV (standard deviation/mean).

### Selection of pilot counties

Hubei and Yunnan were selected as the study provinces because they include two major schistosomiasis-endemic ecological settings in China, namely the lake/marshland setting and the mountainous setting, respectively. Both provinces met the national criteria for transmission interruption in 2020 [29], marking a transition from intense control to more refined management. Pilot counties in each province were selected based on the quality of the surveillance data and expert input, taking into account historical endemicity, recent signals from snail, human, and livestock surveillance, and local control capacity and resource allocation. Ultimately, three counties were selected in Hubei (Xiantao, Qianjiang, and Jiangling) and three in Yunnan (Binchuan, Xiangyun, and Yongsheng). These six pilot counties, spanning two major endemic ecological settings in China, were used to provide an initial field-based assessment of the applicability and stability of the indicator framework under heterogeneous post-transmission-interruption conditions. Field data for the tertiary indicators were collected for the six pilot counties from 2020 to 2024; the operational definitions are provided in Supplementary Table 1.

### Weight calculation

#### Delphi-based weights

Delphi weights were derived from the experts’ importance ratings, adjusted by expert authority, and normalized so that the weights summed to 1 at each level.

For indicator *i*, the authority-adjusted score *S*_*i*_ was calculated as:

$$Si = Ii \times \overline{C}_{{_{r} }} ,$$

where *I*_*i*_ is the mean importance score and ${\overline{C}}_{{r}}$ is the mean authority coefficient.

Within each level, Delphi weights were obtained by normalization:

$$w_{i}^{D} = \frac{ Si }{{ \Sigma Si}}$$

For the hierarchical structure, weights for the lower-level indicators were first normalized within each parent indicator and then multiplied by the parent weight:

$${w}_{level 2 }^{D}= {w}_{parent}^{D} \times Norm({S}_{level 2 |parent}),{ w}_{level 3 }^{D}={ w}_{parent}^{D} \times Norm({S}_{level 3 | parent})$$

where $Norm({S}_{\mathrm{l}\mathrm{e}\mathrm{v}\mathrm{e}\mathrm{l} k | parent})$ denotes the within-parent normalization across subordinate indicators, with $k\in \left\{ 2, 3\right\}$.

#### Entropy weights

The entropy weight method (EWM) is an objective weighting approach that quantifies the information content of an indicator based on its dispersion across observations [30]. Objective weights were estimated using third-level indicator data from the six pilot counties during 2020–2024, capturing each indicator’s relative discriminative power across the sample. To reduce the influence of the distribution of the original data on the entropy calculation, non-normally distributed indicators with positive values were Box–Cox transformed [31]. Indicators containing zeros were transformed using ln(x + 1), whereas approximately normal indicators were left untransformed. All indicators were then min–max normalized according to directionality (positive vs negative).

positive indicator: ${x}_{ij}^{\prime} =\frac{{x}_{ij}-min({x}_{i})}{ max({x}_{i})-min({x}_{i})}$, negative indicator: ${x}_{ij}^{\prime} = \frac{max({x}_{i})-{x}_{ij}}{max({x}_{i})-min({x}_{i})}$

where $i = 1,...,m$ denotes indicators and $j = 1,...,n$ denotes observations; *x*_*ij*_ and *x*_*ij*_^*'*^ denote the original (or transformed) and normalized values, respectively (Supplementary Data 1).

After normalization, the proportion of indicator *i* in observation *j* was calculated as:

$${p}_{ij} = \frac{{x}_{ij}^{\prime}}{{\sum}_{j=1}^{n}{x}_{ij}^{\prime}}$$

The information entropy for indicator *i* was then computed as:

$${e}_{i} = -k\sum_{j=1}^{n}{p}_{ij}ln({p}_{ij}), k = \frac{1}{\mathit{ln}n}$$

Finally, the entropy weight for indicator *i* was obtained as:

$${w}_{i}^{E} =\frac{1 - {e}_{i}}{ \sum_{i=1}^{m}(1 - {e}_{i})}$$

where *n* is the number of observations and *m* is the total number of indicators.

#### Combined weights

In multicriteria decision-making, combining subjective and objective weights allows expert judgement to be integrated with data-driven information, leveraging their complementary strengths [32, 33]. Following the approach used in the Global One Health Intrinsic Drivers Index and the minimum squared-deviation principle, the subjective preference coefficient (*α*) was set to 0.5 [34]. The combined weights for third-level indicators (*w*_*i*_^*c*^) were then calculated as the weighted average of the Delphi and entropy weights (Delphi-EWM):

$${w}_{i}^{c} = \alpha \times {w}_{i}^{D} + (1 - \alpha ) \times {w}_{i}^{E}, \alpha = 0.5$$

### Calculation of the composite risk index (*R*)

The composite risk index (*R*) was used to quantify the risk of schistosomiasis transmission on a scale from 0 to 1, with higher values indicating higher risk. Annual *R* was calculated as the weighted linear aggregation of the normalized third-level indicator values (*x*_*ij*_^*'*^) and their combined weights (*w*_*i*_^*c*^):

$$R =\sum_{i=1}^{n}{w}_{i}^{c} \times {x}_{ij }^{\prime}, R\in [\mathrm{0,1}]$$

### Fuzzy risk grading

Fuzzy set theory addresses the uncertainty at class boundaries by using membership functions to quantify the degree to which an object belongs to a fuzzy set, thereby allowing the logical transition from quantitative measurement to qualitative appraisal [35]. Because mapping a continuous risk measure to discrete risk levels inevitably involves ambiguous boundaries, a trapezoidal membership function (TMF) was used, with core and transition intervals specified to characterize the fluctuations around grade cut-offs, as defined below:

$$\begin{array}{c}\mu (R)=\left\{\begin{array}{cc}0 ,& R\le a \, \mathrm{o}\mathrm{r}\, R\ge \mathrm{d}\\ \frac{R-a}{b-a} ,& a<R\le b\\ 1 ,& b<R\le c\\ \frac{d-R}{d-c} ,& c<R<d\end{array}\right.\end{array}$$

where (*b*,*c*) is the core interval, indicating full membership in the corresponding risk grade, and (*a*,*b*) and (*c*,*d*) are fuzzy transition intervals, which capture the gradual shift between adjacent grades.

In this study, we drew on an established risk level classification [36] and internal team discussions to define five risk levels (very low, low, moderate, high, and very high). Using the grading anchors reported previously [36], the annual composite risk index (*R*) was mapped to these levels, and each level was parameterized with a TMF. The trapezoidal parameters were specified according to a commonly used fuzzy linguistic scaling scheme, ensuring ordered levels, overlap between adjacent intervals, and anchoring at 0 and 1 to reflect the gradual transitions across grade boundaries [37]. To avoid hard threshold cutting and characterize the boundary uncertainty, a transition zone of ± 0.05 was introduced at the boundary of adjacent-level anchor points to determine the trapezoidal parameters (a, b, c, d) of each level (Table 3). Annual risk levels for each pilot county were assigned with the maximum membership principle.

**Table 3:** Risk levels and trapezoidal fuzzy number parameters

Risk level	Trapezoidal fuzzy number (a, b, c, d)
Very low	(0.00, 0.00, 0.15, 0.25)
Low	(0.15, 0.25, 0.35, 0.45)
Moderate	(0.35, 0.45, 0.55, 0.65)
High	(0.55, 0.65, 0.75, 0.85)
Very high	(0.75, 0.85, 1.00, 1.00)

### Robustness assessment

To assess the stability of the results under different balances between subjective and objective weighting, a sensitivity analysis was performed by perturbing the subjective preference coefficient around the baseline (*α* = 0.5). Five scenarios were evaluated (α = 0.3, 0.4, 0.5, 0.6, 0.7), spanning a shift from a greater emphasis on entropy-based weights to a greater emphasis on Delphi-based weights. For each scenario, the *R* values and county–year risk rankings were recalculated.

Robustness was assessed in two dimensions. (1) Distributional assessment: ridgeline plots were used to display the kernel density of the *R* values under each α scenario, allowing the qualitative visual comparison of distributional shape changes under weighting perturbations. (2) Ranking consistency: a 5 × 5 matrix of Spearman’s rank correlation coefficients was computed to quantify the consistency of the county–year risk rankings across scenarios. Correlations closer to 1 indicated lower sensitivity to α and thus the greater robustness of the risk assessment result.

### Statistical analysis

Data from the expert consultation questionnaires and the indicator data for the six pilot counties (2020–2024) were entered and cleaned in Microsoft Excel 2021 (Microsoft Corporation, Redmond, USA). Kendall’s coefficient of concordance (*W*) was calculated using SPSS Statistics 26.0 (IBM Corporation, Armonk, USA). All other analyses were performed in Python 3.13 (Python Software Foundation, Wilmington, DE, USA), including calculation of Delphi metrics (Means, CVs, *C*_*r*_), Box–Cox transformations using SciPy, entropy weight estimation, fuzzy membership grading, and the calculation of the *R*. Robustness analyses were conducted using JoyPy and Pandas to generate ridgeline plots and Spearman rank correlation matrices. A two-sided *P* < 0.05 was considered statistically significant.

## Results

### Characteristics of the participating experts

All invited experts had substantial experience in schistosomiasis control. Most had worked in the field for 20–40 years (80.6%), and the majority were affiliated with the CDC (87.1%). Twenty-one experts (67.7%) held a master’s degree or higher, and twenty-eight (93.4%) held senior professional titles. No experts reported unfamiliarity with the indicators used in this study, indicating a high level of expertise and extensive practical experience (Table 4).

**Table 4:** Demographic and professional information on the experts (*n* = 31)

Subject	Category	*n*	Proportion (%)
Sex	Male	22	71.0
	Female	9	29.0
Age (years old)	30–39	1	3.2
	40–49	12	38.7
	50–59	13	41.9
	≥ 60	5	16.1
Highest degree	Bachelor	10	32.3
	Master	13	41.9
	Doctor	8	25.8
Professional title	Middle	2	6.7
	Deputy Senior	5	16.7
	Senior	23	76.7
Institutional affiliation	CDC	27	87.1
	Health administrative department	1	3.2
	Medical university	2	6.5
	Hospital	1	3.2
Years of engagement in schistosomiasis work	10–20	4	12.9
	20–30	12	38.7
	30–40	13	41.9
	≥ 40	2	6.5
Self-rated familiarity with the topic	Very unfamiliar	0	0.0
	Somewhat unfamiliar	0	0.0
	Neutral	1	3.2
	Familiar	8	25.8
	Extremely familiar	22	71.0

CDC: Centers for Disease Control and Prevention

### Delphi results and indicator refinement

**Fig. 2:**
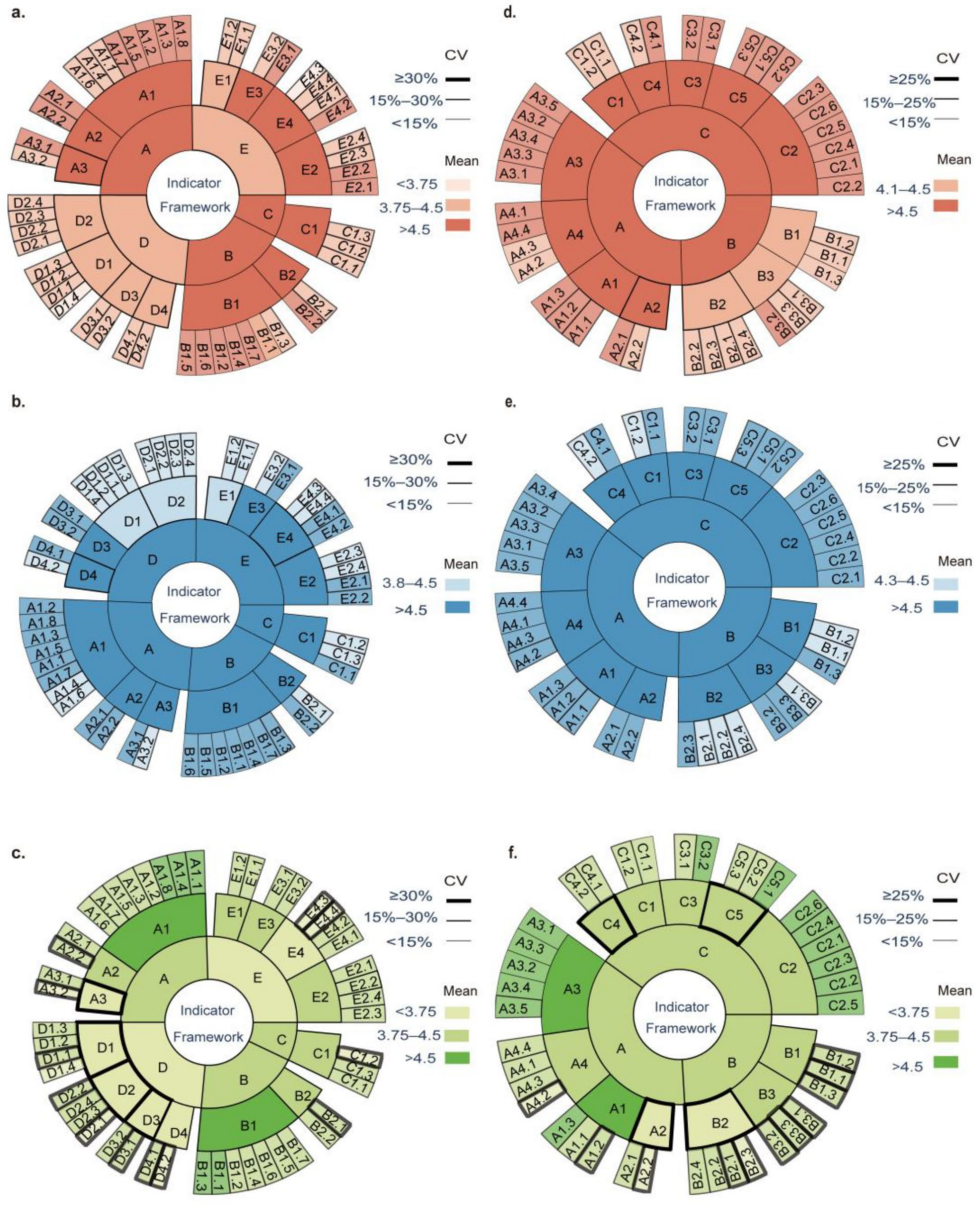
Results of two-round Delphi expert consultation for the multilevel indicator framework. The sunburst charts display a hierarchical structure from inner to outer rings (first-level, second-level, and third-level indicators). Color intensity represents the mean expert rating (Mean), and the line width denotes the coefficient of variation (CV). Panels **a**–**c** present the round 1 results for importance, rationality, and operability, respectively; panels **d**–**f** present the corresponding round 2 results

Two rounds of anonymous Delphi consultation were conducted. The initial framework comprised five first-level, fourteen second-level, and forty-eight third-level indicators. Based on first-round ratings and feedback, the framework was systematically refined: three first-level indicators and one third-level indicator were merged; two second-level and twelve third-level indicators were deleted; and the wording of thirteen indicators was revised. In the second round, all indicators met the prespecified criteria for rationality and importance. A small number of indicators showed comparatively lower operability scores and higher CVs, and four third-level indicators were further refined based on the suggestions of experts. After two iterative rounds, the final transmission risk assessment framework included three first-level, twelve second-level, and thirty-nine third-level indicators. The ratings for rationality, importance, and operability are presented in Fig. 2, and the corresponding data are provided in Supplementary Data 2. The revision process is summarized in Supplementary Table 2.

### Reliability of the Delphi consultation

(1) Expert response. The response rate was 86.1% in round 1 (31/36) and 100.0% in round 2 (31/31).

(2) Authority coefficient. Expert authority remained high across both rounds: *C*_*r*_ ranged from 0.82 to 0.99 in round 1 and from 0.84 to 0.99 in round 2.

(3) Kendall’s *W* for rationality, importance, and operability were 0.197, 0.258, and 0.184, respectively, in round 1, and 0.142, 0.186, and 0.249, respectively, in round 2 (all *P* < 0.001) (Table 5). The CVs ranged from 0 to 37.7% in round 1 and from 0 to 35.1% in round 2 (Fig. 2).

**Table 5:** Degree of coordination across two rounds of Delphi consultation

(Number of) consultations	Evaluation dimensions	Kendall’s *W*	*χ* ^2^ value	*P*-value
Round 1	Rationality	0.197	43.240	< 0.001
	Importance	0.258	527.140	< 0.001
	Operability	0.184	376.434	< 0.001
Round 2	Rationality	0.142	232.516	< 0.001
	Importance	0.186	306.289	< 0.001
	Operability	0.249	408.512	< 0.001

### Weighting results and top-ranked indicators

Based on the Delphi-normalized weights, biological factors received the highest first-level weight (0.350), followed by environmental factors (0.338) and social factors (0.312). The five highest-ranked second-level indicators were climatic factors (0.116), hydrographic factors (0.115), topographic factors (0.107), snail status (0.091), and livestock sources of infection (0.091). Under the combined Delphi-EWM weighting scheme, the third-level indicator weights ranged from 0.0056 to 0.0863. The four highest-ranked third-level indicators were mobile-population surveillance task completion rate (0.0863), area of newly detected snail habitat (0.0846), coverage of snail-related river regulation projects (SRRP) in Yangtze-connected waterways (0.076,1), and military personnel deployed for flood relief (0.0717). Details are provided in Tables 6 and 7.

**Table 6:** Delphi-normalized weights for the first- and second-level indicators

First-level indicators	Delphi weight ${{\boldsymbol{w}}}^{{\boldsymbol{D}}}$	Second-level indicators	Delphi weight ${{\boldsymbol{w}}}^{{\boldsymbol{D}}}$
A. Biological factors	0.350	A1. Livestock sources of infection	0.091
		A2. Wildlife sources of infection	0.078
		A3. Snail status	0.091
		A4. Key populations	0.089
B. Environmental factors	0.338	B1. Climatic factors	0.116
		B2. Topographic factors	0.107
		B3. Hydrographic factors	0.115
C. Social factors	0.312	C1. Economic factors	0.060
		C2. Institutional capacity	0.066
		C3. WASH	0.062
		C4. Implementation of ecological control projects	0.060
		*C5.* Precision control capacity	0.064

**Table 7:** Delphi, entropy, and combined weights of third-level indicators and corresponding county-level data sources

Third-level indicators	Delphi weight ${{\boldsymbol{w}}}^{{\boldsymbol{D}}}$	Entropy weight ${{\boldsymbol{w}}}^{E}$	Combined weight ${{\boldsymbol{w}}}^{C}$	County-level data source (2020–2024)
A1.1. Coverage of fecal examination in local livestock	0.0297	0.0057	0.0177	Animal Disease Prevention and Control Center
A1.2. Coverage of fecal examination in introduced livestock	0.0297	0.0067	0.0182	Animal Disease Prevention and Control Center
A1.3. Positivity rate of wild feces samples	0.0320	0.0000	0.0160	Schistosomiasis Control Institute
A2.1. Infection rate in wild rodents	0.0427	0.0000	0.0213	Schistosomiasis Control Institute
A2.2. Infection status of other wild animals	0.0354	0.0000	0.0177	Schistosomiasis Control Institute
A3.1. Detection rate of existing snail habitats	0.0180	0.0217	0.0199	Schistosomiasis Control Institute
A3.2. Area of newly detected snail habitats	0.0185	0.1507	0.0846	Schistosomiasis Control Institute
A3.3. Area of reemerged snail habitats	0.0184	0.0454	0.0319	Schistosomiasis Control Institute
A3.4. Mean density of live snails	0.0183	0.0294	0.0238	Schistosomiasis Control Institute
A3.5. Nucleic acid-positive snail habitats	0.0178	0.0000	0.0089	Schistosomiasis Control Institute
A4.1. Water-contact exposure rate	0.0237	0.0270	0.0253	Schistosomiasis Control Institute
A4.2. Recreational fishers	0.0209	0.0133	0.0171	Fishing Association or water-contact population survey
A4.3. Migrant construction workers	0.0216	0.0416	0.0316	Bureau of Housing and Urban–Rural Development
A4.4. Military personnel deployed for flood relief	0.0229	0.1205	0.0717	Bureau of Emergency Management
B1.1. Annual mean temperature	0.0387	0.0031	0.0209	Bureau of Meteorological
B1.2. Mean minimum temperature in January	0.0367	0.0058	0.0213	Bureau of Meteorological
B1.3. Annual precipitation	0.0407	0.0077	0.0242	Bureau of Meteorological
B2.1. Elevation	0.0268	0.0266	0.0267	Schistosomiasis Control Institute
B2.2. Vegetation coverage	0.0273	0.0084	0.0179	Schistosomiasis Control Institute
B2.3. Soil moisture	0.0270	0	0.0135	No data source identified
B2.4. Farmland proportion	0.0259	0.0217	0.0238	Bureau of Agriculture and Rural Affairs
B3.1. Density of snail-infested water systems	0.0356	0.0388	0.0372	Schistosomiasis Control Institute
B3.2. Flood events	0.0420	0.0284	0.0352	Bureau of Emergency Management
B3.3. Drought events	0.0375	0.0060	0.0217	Bureau of Emergency Management
C1.1. County fiscal capacity	0.0298	0.0138	0.0218	Bureau of Statistics
C1.2. Per capita income of rural residents	0.0299	0.0062	0.0181	Bureau of Statistics
C2.1. Investment in schistosomiasis control	0.0109	0.0069	0.0089	Bureau of Health
C2.2. Full-time schistosomiasis control staff	0.0113	0.0097	0.0105	Schistosomiasis Control Institute
C2.3. Part-time schistosomiasis control staff	0.0107	0.0152	0.0129	Schistosomiasis Control Institute
C2.4. EQA compliance rate	0.0112	0.0000	0.0056	Schistosomiasis Control Institute
C2.5. Annual professional training sessions	0.0112	0.0155	0.0134	Schistosomiasis Control Institute
C2.6. Flood-season control material stockpile	0.0109	0.0069	0.0089	Schistosomiasis Control Institute
C3.1. Sanitary toilet coverage	0.0314	0.0133	0.0224	Bureau of Agriculture and Rural Affairs
C3.2. Safe water supply coverage	0.0310	0.0000	0.0155	Bureau of Agriculture and Rural Affairs
C4.1. Coverage of SRRP in Yangtze-connected waterways	0.0321	0.1200	0.0761	Schistosomiasis Control Institute
C4.2. Annual growth rate of protected wetland area	0.0274	0.0000	0.0137	No data source identified
C5.1. Mobile-population surveillance task completion rate	0.0219	0.1507	0.0863	Schistosomiasis Control Institute
C5.2. Health education coverage	0.0226	0.0237	0.0231	Schistosomiasis Control Institute
C5.3. Presence of smart sentinel sites	0.0198	0.0099	0.0148	Schistosomiasis Control Institute

SRRP: snail-related river regulation projects; EQA: external quality assessment

### Composite risk index (*R*) results

**Fig. 3:**
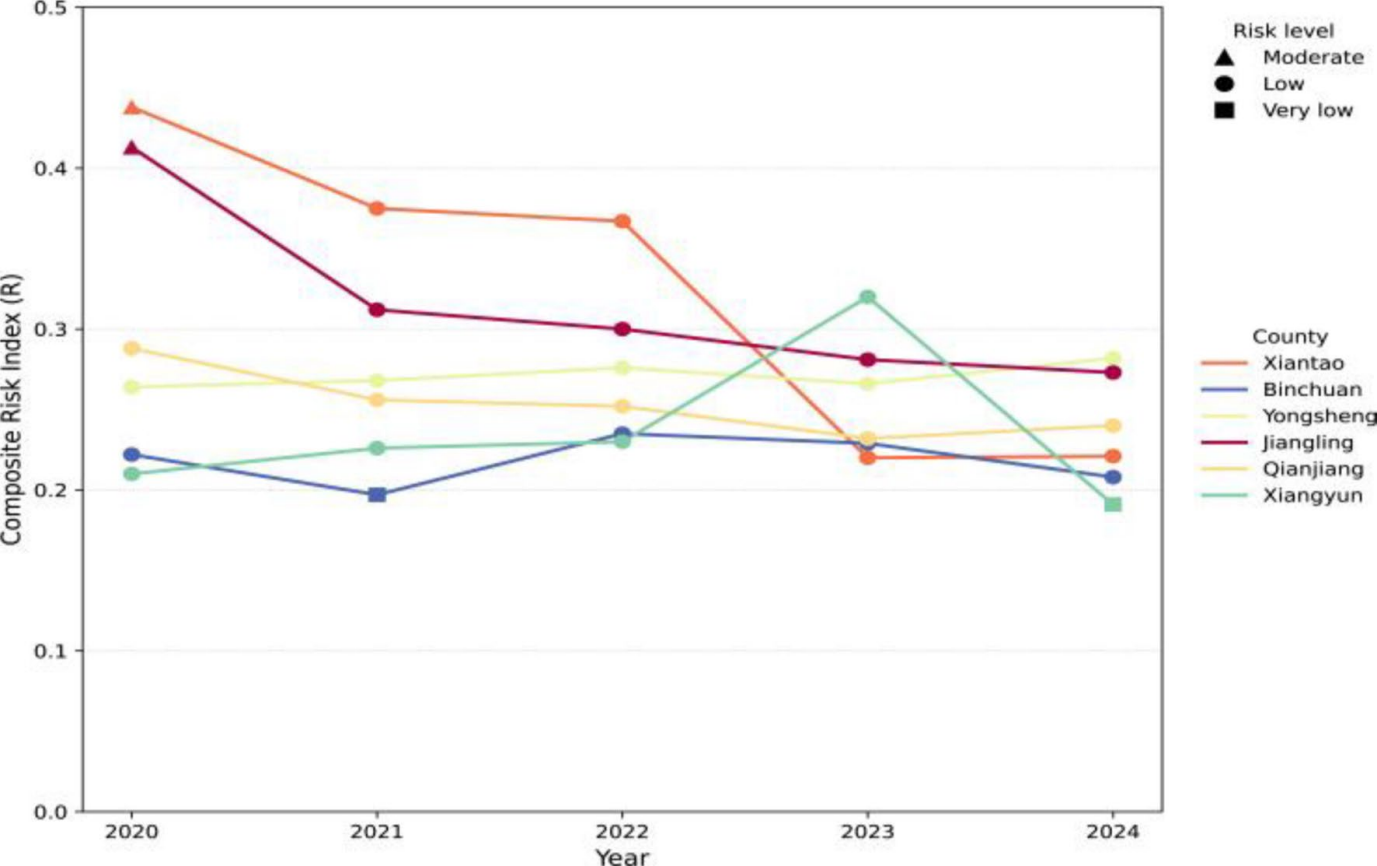
Composite risk index (*R*) values and corresponding fuzzy risk grade classifications for six pilot counties in Hubei and Yunnan, 2020–2024

Across the six pilot counties during 2020–2024, *R* ranged from 0.18 to 0.44. Among the lake/marshland counties in Hubei, Xiantao and Jiangling had relatively high *R* values in 2020 (0.438 and 0.413, respectively), which declined to 0.221 and 0.273 in 2024. Qianjiang ranged from 0.288 in 2020 to 0.240 in 2024. In the mountainous counties of Yunnan, Yongsheng remained relatively stable, ranging from 0.264 in 2020 to 0.282 in 2024. Binchuan showed limited variation, reaching its lowest value of 0.197 in 2021. Xiangyun exhibited greater interannual variability, peaking at 0.322 in 2023 and falling to the lowest observed annual value among all counties, 0.191, in 2024 (Fig. 3).

### Fuzzy risk grading results

During 2020–2024, the six pilot counties were predominantly classified as low risk of schistosomiasis transmission (Fig. 3). Xiantao and Jiangling were classified as moderate risk in 2020 and low risk thereafter, whereas Qianjiang was classified as low risk throughout the study period. Yongsheng was classified as low risk in all years, and Binchuan was classified as very low risk in 2021 but as low risk in the remaining years. Xiangyun remained classified as low risk from 2020 to 2023, despite an increase in *R*, because its scores fell within the core or transition intervals of low risk. It was classified as very low risk in 2024.

### Robustness to weighting perturbations

**Fig. 4:**
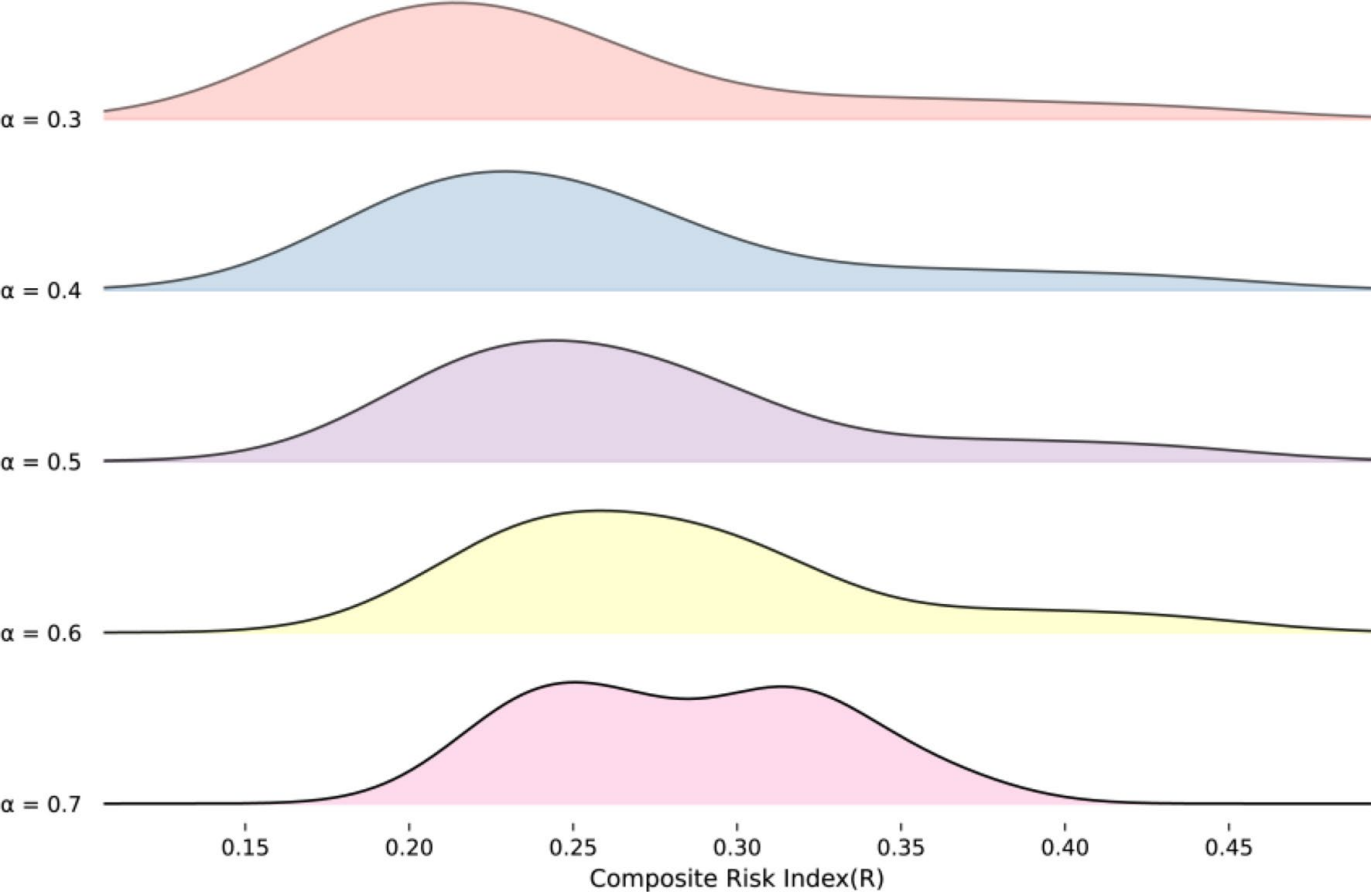
Distribution of the composite risk index (*R)* values under different α weighting scenarios (α = 0.3–0.7)

**Fig. 5:**
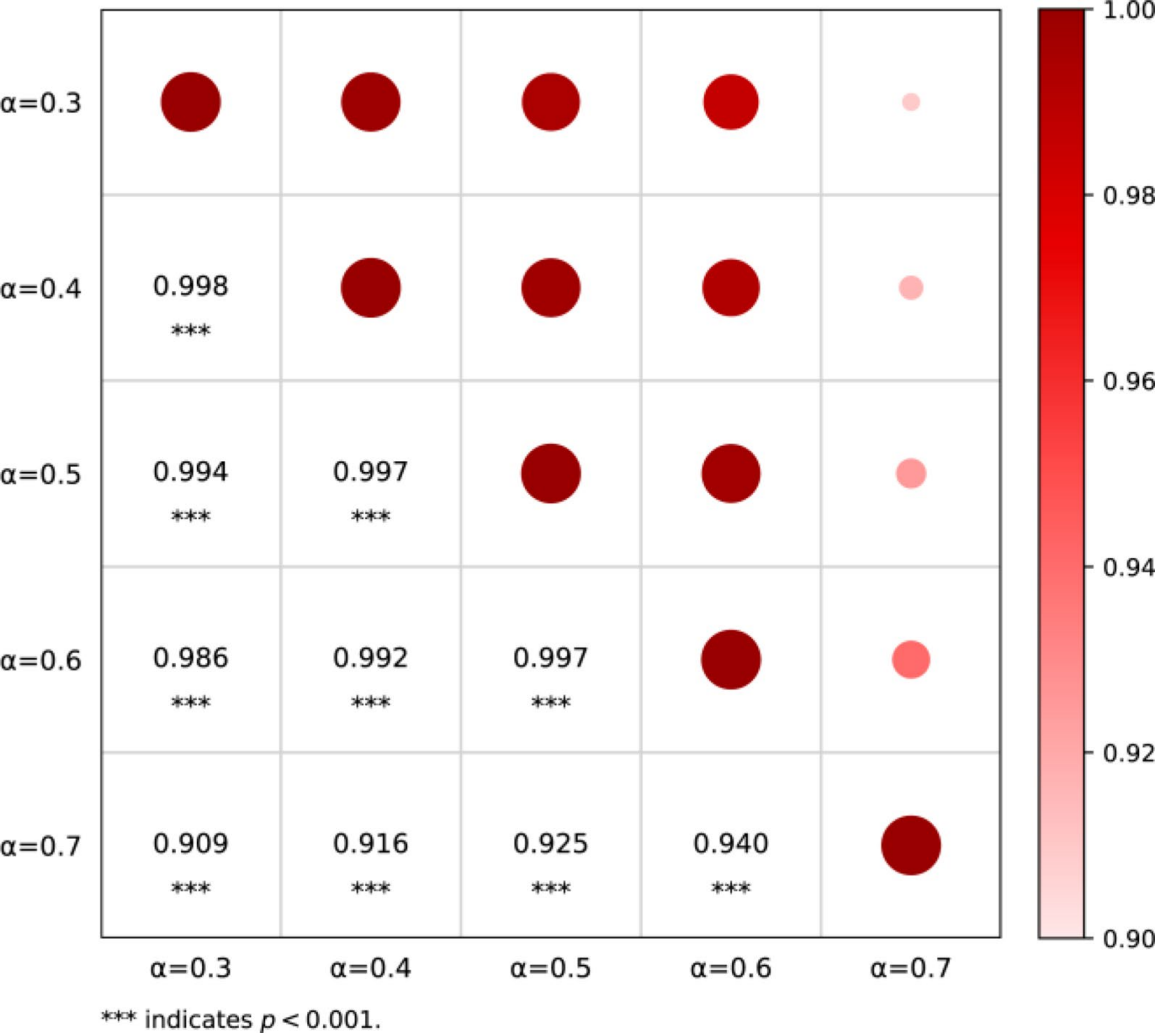
Pairwise consistency matrix of county-level risk rankings under different α weighting scenarios (α = 0.3–0.7)

Ridgeline plots showed broadly similar distributions of *R* across the five α scenarios (0.3–0.7), with substantial overlap in the high-density ranges, where a modest change in shape was observed at α = 0.7 (Fig. 4). Spearman’s rank correlations of county risk rankings were high across all pairwise comparisons among the five α scenarios (*ρ* = 0.909–0.998, all *P* < 0.001), with slightly lower agreement in comparisons involving α = 0.7 (*ρ* = 0.909–0.940) (Fig. 5). Overall, both the distribution of *R* and county–year risk rankings were stable under perturbations of the subjective preference coefficient.

## Discussion

An initial assessment framework was developed by synthesizing evidence from previous studies, national surveillance requirements for schistosomiasis control, and multisectoral policy documents. Two rounds of anonymous Delphi consultation were conducted to refine the framework and improve its scientific rigor and relevance to the post-transmission-interruption context. The response rates were 86.1% and 100.0% in the two rounds, respectively. The expert authority coefficients remained high and increased in round 2, supporting the credibility of the consultation process. Kendall’s *W* scores were statistically significant (all *P* < 0.001), indicating that the expert opinions showed good consistency. The final indicator framework for the assessment of the schistosomiasis transmission risk comprised three dimensions: biological, environmental, and social.

In terms of the biological factors, livestock sources of infection and snail status received the highest weights (both 0.091), underscoring their relative importance in sustaining schistosomiasis transmission. Bovines are major reservoir hosts of *S. japonicum* and may continue to exert infection pressure, given the restocking in some lake and marshland areas and the practical difficulty of phasing them out in mountainous settings [38]. This pressure may be further amplified by gaps in the regulation of inter-regional livestock movements [39], and the resulting latent risk may not be readily detected by routine surveillance. As the sole intermediate host of *S. japonicum*, *O. hupensis* has been the focus of sustained control. However, the total area of suitable snail habitat is still estimated to be approximately 3.6 billion square meters [14]. Therefore, the persistence and extent of this habitat remain fundamental biological challenges for risk prevention and management.

Among the environmental factors, climatic factors (0.116) and hydrographic factors (0.115) carried relatively high weights, reflecting their important roles in shaping the stability of schistosomiasis transmission. Because snails thrive under warm and humid conditions, changes in temperature and rainfall can affect their survival, reproduction, and spatial distribution [21]. Previous studies suggested that warming may be linked to a northward expansion of climatically suitable areas for snails beyond the Yangtze River [22], whereas increased precipitation and wetter environments may promote population persistence and recovery [40]. Moreover, extreme events may further elevate the risk by amplifying water-level fluctuations and hydrological connectivity, facilitating the dispersal and recolonization of snail habitats [41]. Consistent with this, after the Yangtze River Basin floods in 2020, snails were detected in potentially suitable habitats in Jiangxi and Anhui, supporting the roles of climatic and hydrological anomalies in reshaping transmission risk patterns [42].

At the current stage of schistosomiasis control, residual transmission risk may become increasingly difficult to detect and more susceptible to the cumulative effects of sociostructural vulnerabilities [43]. Persistently low endemicity can erode public risk perception [44], whereas resource constraints and competing public health priorities may lead to fluctuations in frontline investment and surveillance capacity [45]. As a result, weaknesses in routine program delivery may become potential triggers for the re-establishment of transmission chains. Process- and governance-oriented indicators had relatively high weights, including the mobile-population surveillance completion rate (0.0863) and SRRP coverage in Yangtze-connected waterways (0.0761), highlighting the control system’s capacity to respond to external disturbances. Field investigations provided illustrative examples: mobile-population surveillance in Jiangling was disrupted in 2020 by COVID-19, and some snail-infested waterway management projects were delayed during 2020–2021 amid funding fluctuations. Taken together, these observations point to vulnerabilities in the continuity and implementation stability of frontline control programs.

Notably, several contextual and infrastructural indicators showed limited variation between pilot counties. This pattern appeared to reflect ceiling effects arising from consistently achieved program targets (e.g., safe water supply), persistently zero detection of certain animal-host indicators (e.g., wild rodents), and a paucity of field data on specific ecological measures (e.g., wetland area change). Consequently, the information entropy for these indicators was zero. Relying solely on the EWM would marginalize or even exclude these factors from the evaluation framework. To address this issue, an equal-ratio Delphi-EWM scheme was used. This approach retained data-driven information while incorporating expert judgment to compensate for limited objective dispersion, thereby avoiding undue structural simplification of the indicator framework.

The fuzzy grading results indicated that the six counties were generally classified as having a low risk of schistosomiasis transmission during the assessment period, consistent with the epidemiological profile of China during the post-transmission-interruption stage of disease control [38]. Furthermore, high ranking concordance under α perturbations supported the robustness of the assessment. At the same time, the observed differences in *R* values can be interpreted as the combined effects of multiple weighted dimensions of residual transmission risk, rather than the influence of any single indicator alone. In the lake/marshland counties, Xiantao and Jiangling had relatively high *R* values, driven by heavily weighted indicators with high observed values, including dense water networks, extensive snail habitats, high densities of living snails, and frequent population movement. By contrast, Binchuan and Xiangyun had lower baseline *R* values, but their interannual fluctuations were more pronounced on a relative scale, although the absolute changes remained modest. In these mountainous counties, such variation appeared to be related to climatic variability and the re-emergence or newly detected snail habitats during the study period.

Taken together, the findings suggest that transmission risk can be differentiated across heterogeneous ecological settings with this assessment framework, supporting its practical utility and robustness. Importantly, the *R* should be interpreted as a relative measure of residual transmission risk at the county level under post-transmission-interruption conditions, rather than as a direct measure of ongoing infection events. By integrating biological, environmental, and social dimensions, the framework translates multidimensional signals into an interpretable county-level risk profile. In China’s post-transmission-interruption stage of schistosomiasis control, the framework provides an actionable quantitative basis for decision-making by identifying key drivers, enabling risk stratification, and informing more efficient allocation of public health resources to consolidate long-term control gains.

Several limitations should be acknowledged. First, indicators with limited between-county variability may have been underweighted, particularly those capturing wildlife-host infections and certain ecological measures. Second, although the framework showed good discrimination and stability in the six pilot counties, the present study should be regarded as a pilot field-based application and preliminary validation rather than full external validation in the strict methodological sense. Including six counties from two major endemic ecological settings in China allowed an initial assessment of the framework’s applicability across heterogeneous transmission environments. However, further external validation, evaluation, and applicability assessment of the framework are needed across a broader range of endemic counties, including areas with poor data quality. Finally, given the dynamic nature of the transmission risk, the periodic recalibration of indicator specifications and weights using routine surveillance and program data may be required to maintain the sensitivity and timeliness of the framework.

## Conclusions

A county-level indicator framework was developed to assess the risk of schistosomiasis transmission in China’s post-transmission-interruption stage of disease control, integrating Delphi consultation, the entropy weight method, and fuzzy grading. When applied to six pilot counties (2020–2024), the framework yielded low overall risk classifications (*R*: 0.18–0.44), while still characterizing cross-ecological differences between lake/marshland and mountainous settings. The risk rankings remained highly consistent under perturbations of the subjective preference coefficient, supporting the robustness of the assessment outputs. Therefore, this framework provides an operational tool to support the prioritization of risk-informed surveillance and the more-efficient allocation of control resources in low-endemicity contexts.

## Supplementary Information

Additional file 1: Original Chinese questionnaire used in the Delphi consultation

Additional file 2: Supplementary Table 1. Operational definitions, calculation methods, units, and directionality of the final third-level indicators in the county-level schistosomiasis transmission risk assessment framework

Additional file 3: Supplementary Data 1. Standardized indicator value matrix for the six pilot counties, 2020–2024

Additional file 4: Supplementary Data 2. Expert Rating Form

Additional file 5: Supplementary Table 2. Two rounds of Delphi consultation and indicator refinement

## Data Availability

The full study protocol and the datasets, are available, following manuscript publication, upon request from the corresponding author.

## Abbreviations

α: The subjective preference coefficient
Ca: The judgement basis
CCDC: Chinese Center for Disease Control and Prevention
CDC: Centers for Disease Control and Prevention
COVID-19: Coronavirus disease 2019
Cr: The authority coefficient
Cs: The familiarity coefficient
CV: The coefficient of variation
EQA: External quality assessment
EWM: The entropy weight method
*R*: The composite risk index
SRRP: Snail-related river regulation projects
WHO: World Health Organization
