# Supplementary material for: A county-level indicator framework for assessing schistosomiasis transmission risk in post-transmission-interruption China
Source: Infect Dis Poverty. 2026 May 18;15:58. doi: 10.1186/s40249-026-01453-6 (PMC13182038; doi:10.1186/s40249-026-01453-6)
Supplement: Supplementary file 1 — Additional file 1: Original Chinese questionnaire used in the Delphi consultation [file 40249_2026_1453_MOESM1_ESM.docx]

**血吸虫病传播风险评估指标体系构建专家咨询问卷**

**填写说明**

本指标框架分为三级，包含5个一级指标，14个二级指标和48个三级指标，指标概览附于填写说明后。填写时请注意：每个指标从合理性、重要性、数据可及性、判断依据以及熟悉程度五个方面进行评价：

1. **合理性**(1-5)指所选指标能够准确地反映某一方面，不脱离实际。以5分计，非常合理计5分，较合理计4分，一般计3分，不太合理计2分，很不合理计1分。
2. **重要性**(1-5)指所选指标是在评价某一方面时较为公认的重要的指标。以5分计，非常重要计5分，较重要计4分，一般重要计3分，不太重要计2分，极不重要计1分。
3. **可操作性**(1-5)指所选指标的可获得性与收集的难易程度，能尽可能利用现有报告资料。以5分计，易操作计5分，比较容易操作计4分，一般计3分，较 难操作计2分，很难操作计1分。
4. **判断依据**是指专家对各具体指标的重要性、可操作性等特征进行判断时的主要依据。分为“实践经验”、“理论依据”、“同行了解”以及“直觉”四个方面，按照各方面的依据对专家的影响程度大小进行评分，以3分计，影响较大计3分，一般计2分，影响较小计1分。每个空格均需打分。
5. **熟悉程度**是评判专家对该指标所评价内容的熟悉程度。其量化分值分别为：非常熟悉为 1.0 分，较熟悉为 0.8 分，一般为 0.6 分，不太熟悉为 0.4 分，非常不熟悉为 0.2 分。为方便专家作答，问卷填写时采用 5、4、3、2、1 的评分方式，后续统计分析时再对应换算为 1.0、0.8、0.6、0.4 和 0.2。
6. 开放性建议：若您对指标分类、名称、释义及计算公式等若有不同看法，您可指出（如建议删除或需要修改名称），并在**修改意见**处说明；每个评价表后附有留白处，您可将建议添加的指标直接用红色添加。
7. 基本情况表：问卷最后附有专家基本情况表，请您按照自身情况填写，以便我们做进一步分析。

Expert consultation questionnaire on the development of the schistosomiasis transmission risk assessment indicator framework

Instructions for Completion

This indicator framework has a three-level structure and includes 5 first-level indicators, 14 second-level indicators, and 48 third-level indicators. An overview of the indicators is provided after these instructions. When completing the questionnaire, please evaluate each indicator from five aspects: rationality, importance, operability, judgement basis, and familiarity.

（1）Rationality (1–5):Rationality refers to whether the selected indicator can accurately reflect a given aspect and is consistent with practical conditions. Scoring was based on a five-point scale: very rational = 5, relatively rational = 4, average = 3, somewhat irrational = 2, and very irrational = 1.

（2）Importance (1–5):Importance refers to whether the selected indicator is widely regarded as important for evaluating a given aspect. Scoring was based on a five-point scale: very important = 5, relatively important = 4, average = 3, somewhat unimportant = 2, and not important at all = 1.

（3）Operability (1–5):Operability refers to the accessibility of the selected indicator and the ease of data collection, with preference given to indicators that can be obtained from existing reporting materials. Scoring was based on a five-point scale: very easy to operate = 5, relatively easy to operate = 4, average = 3, relatively difficult to operate = 2, and very difficult to operate = 1.

（4）Judgement basis:Judgement basis refers to the main basis on which experts evaluated the characteristics of each indicator, such as importance and operability. It includes four aspects: practical experience, theoretical evidence, peer awareness, and intuition. Each aspect was scored according to its degree of influence on the expert’s judgement using a three-point scale: large influence = 3, medium influence = 2, and small influence = 1. A score was required for each item.

（5）Familiarity:Familiarity refers to the degree to which the expert is familiar with the content covered by each indicator. The corresponding quantitative scores were defined as follows: very familiar = 1.0, relatively familiar = 0.8, average = 0.6, somewhat unfamiliar = 0.4, and very unfamiliar = 0.2. For ease of questionnaire completion, experts rated familiarity on a 5-point scale (5, 4, 3, 2, and 1), which was subsequently converted to 1.0, 0.8, 0.6, 0.4, and 0.2, respectively, during data analysis.

（6）Open-ended suggestions:If you have different views on the classification, name, definition, or calculation formula of any indicator, please indicate them in the space for revision comments (for example, if you suggest deleting an indicator or revising its name). Blank space is provided after each evaluation form, and any suggested new indicators may be added directly in red text.

（7）Basic information form:A form on experts’ basic information is attached at the end of the questionnaire. Please complete it according to your own situation to facilitate further analysis.

**第一轮**

**表1** **血吸虫病传播风险评估一级指标评分表**

| **一级指标名称** | **一级指标定义** | **计算公式（单位）** | **合理性** | **重要性** | **可操作性** | **判断依据（1-3）** | | | | **熟悉程度** | **对一级指标修改意见** |
| --- | --- | --- | --- | --- | --- | --- | --- | --- | --- | --- | --- |
|  |  |  | **（1-5）** | **（1-5）** | **（1-5）** | **实践经验** | **理论依据** | **同行了解** | **直**  **觉** | **（1-5）** | **（指标名称、释义、分类及计算公式等）** |
| A 传染源 | 传染性疾病流行的三个基本环节之一。传染源是血吸虫病传播的源头，包括感染血吸虫病的人类、家畜和野生动物等。通过监测传染源的感染状态和传播风险，可有效评估疫情扩散潜力并制定针对性防控措施 | = $\sum_{k=1}^{n=3} Ak^{'}权重$ |  |  |  |  |  |  |  |  |  |
| B 传播途径 | 血吸虫病通过接触含有尾蚴的水体传播。尾蚴从钉螺体内释放到水体中，人或动物接触疫水时尾蚴侵入皮肤，完成传播链。传播途径监测需聚焦钉螺孳生环境（尾蚴来源）和疫水暴露风险（传播媒介）两大核心环节 | = $\sum_{k=1}^{n=2} Bk^{'}权重$ |  |  |  |  |  |  |  |  |  |
| C 易感人群 | 血吸虫病在流行区的人群中普遍易感。鉴于当前处于低流行状态，在血吸虫病传播链中，那些因职业、行为或流动性特征而接触疫水风险显著高于一般人群的群体，需要我们重点关注 | = $\sum_{k=1}^{n=1} Ck^{'}权重$ |  |  |  |  |  |  |  |  |  |
| D 自然环境因素 | 影响血吸虫病传播的各类自然环境要素及相关干预工程的统称。涵盖了气候、地形、水文、自然灾害等自然条件的被动调节作用，以及生态调控工程等人为措施对环境结构与功能的主动调节作用。该类因素通过影响钉螺孳生地和疫水暴露环境，在血吸虫病传播链中构成重要的物理和生态调控基础 | = $\sum_{k=1}^{n=4} Dk^{'}权重$ |  |  |  |  |  |  |  |  |  |
| E 社会因素 | 影响血吸虫病传播的社会要素与主动防控措施的集合体，通过调控经济资源配置、机构效能、卫生设施水平及精准干预手段，动态管理人群活动与疫水暴露风险，同时在防控体系薄弱环节识别潜在风险点，构成传播链中社会与人文维度的调控基础 | = $\sum_{k=1}^{n=4} Ek^{'}权重$ |  |  |  |  |  |  |  |  |  |
| 对一级指标的增删意见（若需要增加指标，请注明增加指标的名称及其定义）： | | | | | | | | | | | |

**表2 血吸虫病传播风险评估二级指标评分表**

| **一级指标名称** | **二级指标名称** | **二级指标定义** | **计算公式（单位）** | **合理性** | **重要性** | **可操作性** | **判断依据（1-3）** | | | | **熟悉程度** | **对二级指标修改意见** |
| --- | --- | --- | --- | --- | --- | --- | --- | --- | --- | --- | --- | --- |
|  |  |  |  | **（1-5）** | **（1-5）** | **（1-5）** | **实践经验** | **理论依据** | **同行了解** | **直**  **觉** | **（1-5）** | **（指标名称、释义、分类及计算公式等）** |
| A 传染源 | A1人群病情 | 反映人类群体感染血吸虫病的现状及动态变化，重点关注当地常住人口和流动人口的感染风险 | = $\sum_{i=1}^{n=8} A1.i^{'}权重$ |  |  |  |  |  |  |  |  |  |
|  | A2家畜病情 | 家畜（如牛、羊等终宿主）作为血吸虫病传染源的综合风险，涵盖其感染状态（感染率）及当前暴露风险的动态变化趋势（有螺区动态存栏数）。通过传播链，直接或间接影响疫情传播的潜在强度。 | = $\sum_{i=1}^{n=2} A2.i^{'}权重$ |  |  |  |  |  |  |  |  |  |
|  | A3野生动物感染情况 | 野生动物（如野鼠、麋鹿等）是钉螺的自然宿主，其感染率反映生态传播风险 | = $\sum_{i=1}^{n=2} A3.i^{'}权重$ |  |  |  |  |  |  |  |  |  |
| B 传播途径 | B1 螺情 | 钉螺是血吸虫病的唯一中间宿主，其分布密度和感染状态直接决定尾蚴释放量。通过监测钉螺环境，评估尾蚴来源风险 | = $\sum_{i=1}^{n=7} B1.i^{'}权重$ |  |  |  |  |  |  |  |  |  |
|  | B2水体及环境污染状况 | 通过监测水体环境中尾蚴分布情况和水体周围野粪中的血吸虫虫卵分布的动态变化，评估水体感染强度及粪便污染对血吸虫病传播的驱动作用，定位传播链的上游污染源（野粪输入）和下游传播风险（疫水暴露） | = $\sum_{i=1}^{n=2} B2.i^{'}权重$ |  |  |  |  |  |  |  |  |  |
| C 易感人群 | C1重点人群 | 根据《全国血吸虫病监测方案》以及《加快实现消除血吸虫病目标行动方案》，将易感人群按职业、行为或流动性特征聚焦于农民/渔船民，休闲垂钓人员以及重大工程外来施工人员 | = $\sum_{i=1}^{n=3} C1.i^{'}权重$ |  |  |  |  |  |  |  |  |  |
| D 自然环境因素 | D1气候因素 | 通过温度、降水、湿度等气象条件直接影响钉螺生命周期与疫水暴露风险的环境变量 | = $\sum_{i=1}^{n=4} D1.i^{'}权重$ |  |  |  |  |  |  |  |  |  |
|  | D2地形因素 | 通过土壤、高程、植被等地理特征间接调控钉螺分布，决定传播路径的易感性 | = $\sum_{i=1}^{n=4} D2.i^{'}权重$ |  |  |  |  |  |  |  |  |  |
|  | D3自然灾害 | 通过洪涝、干旱等极端事件短期内剧烈改变钉螺孳生环境，直接冲击传播链稳定性 | = $\sum_{i=1}^{n=2} D3.i^{'}权重$ |  |  |  |  |  |  |  |  |  |
|  | D4生态防控工程实施情况 | 通过水利工程、湿地修复等生态工程措施，主动调控钉螺孳生环境，阻断血吸虫病传播链的实施成效评估 | = $\sum_{i=1}^{n=2} D4.i^{'}权重$ |  |  |  |  |  |  |  |  |  |
| E 社会因素 | E1 经济 | 通过县级财政水平与农村居民人均收入两个维度，评估血吸虫病防控工作中经济资源的可及性与个体行为的经济驱动能力，为防螺工程、健康教育及医疗保障等防控措施提供经济支撑 | = $\sum_{i=1}^{n=2} E1.i^{'}权重$ |  |  |  |  |  |  |  |  |  |
|  | E2机构能力建设 | 通过疾控机构网络覆盖、专业人员配置及应急响应效能，保障防控措施的执行力度 | = $\sum_{i=1}^{n=4} E2.i^{'}权重$ |  |  |  |  |  |  |  |  |  |
|  | E3水和卫生设施 | 安全饮水、普及卫生厕所是减少疫水接触风险，阻断传播途径的有效措施 | = $\sum_{i=1}^{n=2} E3.i^{'}权重$ |  |  |  |  |  |  |  |  |  |
|  | E4精准防控 | 运用数据驱动的风险识别、靶向干预手段及智能技术应用，对血吸虫病传播链尚存的关键节点（现存病例、高危行为、钉螺潜在孳生区域）实施分级管理与精准阻断的防控 | = $\sum_{i=1}^{n=4} E4.i^{'}权重$ |  |  |  |  |  |  |  |  |  |
| 对二级指标的增删意见（若需要增加指标，请注明增加指标的名称及其定义）： | | | | | | | | | | | | |

**表3 血吸虫病传播风险评估三级指标评分表**

| **一级指标名称** | **二三级指标名称** | **三级指标定义** | **计算公式（单位）** | **合理性** | **重要性** | **可操作性** | **判断依据（1-3）** | | | | **熟悉程度** | **对三级指标修改意见** |
| --- | --- | --- | --- | --- | --- | --- | --- | --- | --- | --- | --- | --- |
|  |  |  |  | **（1-5）** | **（1-5）** | **（1-5）** | **实践经验** | **理论依据** | **同行了解** | **直**  **觉** | **（1-5）** | **（指标名称、释义、分类及计算公式等）** |
| **A传染源** | **A1人群病情** | | | | | | | | | | | |
|  | A1.1本地居民血清阳性率 | 在本年度内，血吸虫病流行县开展本地居民血吸虫病现场流行病学监测调查，先后经过免疫学和病原学检查均发现为阳性结果的比例 | （病原学检查阳性人数/进行病原学检查人数）/（进行免疫学检查人数÷免疫学检查阳性人数）*100（%） |  |  |  |  |  |  |  |  |  |
|  | A1.2本地居民粪检阳性率 | 在本年度内，血吸虫病流行区开展本地居民血吸虫病病原学检查且结果为阳性的比例 | 当地居民粪检阳性人数/经病原学检查的当地居民人数*100（%） |  |  |  |  |  |  |  |  |  |
|  | A1.3急性血吸虫病病例数 | 在本年度内，经各级各类医疗卫生机构诊断报告并核实确认的急性血吸虫病（是血吸虫病的一种临床类型，主要由于人在短期内一次感染或再次感染大量血吸虫尾蚴而引发）病例总人数 | 本年内经核实确认的急性血吸虫病例数（例） |  |  |  |  |  |  |  |  |  |
|  | A1.4新发现晚期血吸虫病病例 | 在本年度内，经各级各类医疗卫生机构通过诊断首次确诊为晚期血吸虫病的患者病例数量 | 本年内首次确诊为晚期血吸虫病病例数（例） |  |  |  |  |  |  |  |  |  |
|  | A1.5新发慢性血吸虫病病例数 | 在本年度内，经各级各类医疗卫生机构通过诊断首次确诊为慢性血吸虫病的患者病例数量 | 本年内首次确诊为慢性血吸虫病病例数（例） |  |  |  |  |  |  |  |  |  |
|  | A1.6流动人群血检阳性率 | 在本年度内，采用主动监测与被动监测相结合的方式对来自和往返血吸虫病流行区的人员开展调查。其中血吸虫病免疫学检查结果为阳性所占的比例 | 流动人群血检阳性人数/流动人群免疫学检查总人数*100（%） |  |  |  |  |  |  |  |  |  |
|  | A1.7流动人群粪检阳性率 | 在本年度内，针对输入性人群进行血吸虫病病原学检查且结果为阳性的比例 | 流动人群病原学检查阳性人数/流动人群病原学检查总人数*100（%） |  |  |  |  |  |  |  |  |  |
|  | A1.8人群感染率 | 在本年度内，通过血清学和粪便检查确诊为血吸虫病的人数占被检查总人数的比例 | 确诊为血吸虫病的人数/被检查总人数*100（%） |  |  |  |  |  |  |  |  |  |
|  | **A2家畜病情** | | | | | | | | | | | |
|  | A2.1本地家畜粪检阳性数 | 在本年度内，县级疾病预防控制机构会同当地动物疫病防控机构以监测范围内的本地家畜（牛、羊等）为对象，采用病原学（毛蚴孵化法）检测家畜血吸虫感染情况 | 经病原学检查发现当地家畜粪检阳性头数（头） |  |  |  |  |  |  |  |  |  |
|  | A2.2引进家畜感染情况 | 从血吸虫病流行区引进的家畜（牛、羊等）由于运输、检疫等因素影响，可能成为重要输入性传染源。依照监测方案，采用病原学（毛蚴孵化法）检测引进家畜血吸虫感染情况 | 经病原学检查发现引进家畜粪检阳性头数（头） |  |  |  |  |  |  |  |  |  |
|  | **A3野生动物感染情况** | | | | | | | | | | | |
|  | A3.1野鼠感染率 | 野鼠监测是风险监测的重要一环，说明其在低流行态势下可能是重要的传染源之一。对人、畜可及的有螺环境或可疑钉螺孳生环境捕捉野鼠，并通过实验室检测了解该年度野鼠血吸虫感染情况 | 检测出阳性的野鼠只数/检测的野鼠总只数*100% |  |  |  |  |  |  |  |  |  |
|  | A3.2其它野生动物感染情况 | 由于生态保护、恢复计划的实施，野生动物在优化的生态环境中活动，可能成为血吸虫病的潜在传染源。在野鼠监测过程中若有发现野生动物的感染情况，可以及时发现并评估其对血吸虫病传播的风险 | 是否发现有感染血吸虫病的野生动物（是=1/否=0） |  |  |  |  |  |  |  |  |  |
| **B传播途径** | **B1螺情** | | | | | | | | | | | |
|  | B1.1现有钉螺覆盖比 | 指县级行政区域内，钉螺分布的总面积占该县行政总面积的百分比。反映钉螺在县域内的空间分布集中度 | 该县现有钉螺总面积（m^2^）/该县行政总面积（m^2^）*100% |  |  |  |  |  |  |  |  |  |
|  | B1.2新发现钉螺面积 | 根据监测方案对监测范围内螺情调查时，发现历史上从未出现钉螺的环境中，首次查到有钉螺分布的钉螺孳生环境面积 | 统计本年度首次查到有钉螺分布的孳生环境面积（m^2^） |  |  |  |  |  |  |  |  |  |
|  | B1.3复现钉螺面积 | 在历史有螺环境内，经防治曾被确认消灭，但连续两年及以上再度查到有活螺分布的钉螺孳生环境面积 | 统计本年度复现的钉螺孳生环境面积（m^2^） |  |  |  |  |  |  |  |  |  |
|  | B1.4活螺平均密度 | 根据监测方案，每年度在监测范围内调查获得每单位面积（通常为0.1平方米）中活钉螺的数量 | 捕获活螺数/调查框数（0.1/m^2^） |  |  |  |  |  |  |  |  |  |
|  | B1.5感染性钉螺密度 | 根据监测方案，每年度在监测范围内调查获得每单位面积（通常为0.1平方米）中感染血吸虫的活钉螺的数量 | 感染螺数/调查框数（0.1/m^2^） |  |  |  |  |  |  |  |  |  |
|  | B1.6钉螺感染率 | 通过监测调查获得的钉螺中，通过镜检或LAMP（环介导等温扩增技术？）检测出感染血吸虫的钉螺所占的比例 | 感染螺数/解剖螺数*100（%） |  |  |  |  |  |  |  |  |  |
|  | B1.7活螺框出现率 | 在调查的框数中，发现活钉螺的框数所占的比例，用于评估钉螺在调查区域内的分布范围 | 活螺框数/调查框数*100（%） |  |  |  |  |  |  |  |  |  |
|  | **B2水体及环境污染状况** | | | | | | | | | | | |
|  | B2.1哨鼠阳性（水体感染）率 | 在血吸虫病流行区的重点水域，对环境水体中的尾蚴检测常采用哨鼠感染法，尤其在感染率低或感染性钉螺分布广的地区更敏感。 | 阳性哨鼠数/解剖哨鼠总数*100（%） |  |  |  |  |  |  |  |  |  |
|  | B2.2野粪阳性率 | 在人畜活动频繁的有螺环境中采集的哺乳动物（牛、羊、猪、马属、狗等）粪便样本中，检测到血吸虫虫卵的阳性比例，用以追踪血吸虫病传播链中野粪污染水源的风险 | 阳性野粪数/采集野粪总数*100（%） |  |  |  |  |  |  |  |  |  |
| **C易感人群** | **C1重点人群** | | | | | | | | | | | |
|  | C1.1高暴露职业人群（农民/渔船民）比例 | 在血吸虫病流行区域内，从事水稻种植、水产养殖、船舶驾驶等高暴露职业的常住及流动人口占总人口的比例 | （农民人数+渔船民人数）/总人口*100（%） |  |  |  |  |  |  |  |  |  |
|  | C1.2休闲垂钓人员数 | 在疫区水体（如湖泊、池塘、沟渠等）进行垂钓的人员数量，按人次统计（同一人多次垂钓计多次） | 统计垂钓的人员数量(人次/月) |  |  |  |  |  |  |  |  |  |
|  | C1.3重大工程外来施工人员数 | 在血吸虫病流行区内，因基建、水利、能源等重大项目需短期集中居住（≥3个月）的外地务工人员总数 | 各项目合同外地务工人员总数（人） |  |  |  |  |  |  |  |  |  |
| **D 自然环境因素** | **D1 气候因素** | | | | | | | | | | | |
|  | D1.1 年平均气温 | 年平均气温决定区域是否具备钉螺长期存活条件，且血吸虫尾蚴在水中存活、释放均受温度影响 | 统计全年气温的平均值（℃） |  |  |  |  |  |  |  |  |  |
|  | D1.2 一月平均最低气温 | 熟一月通常是冬季最冷月份，低温会使钉螺进入休眠，而极端低温可直接杀死钉螺成体及虫卵 | 统计一月份每日最低气温的平均值（℃） |  |  |  |  |  |  |  |  |  |
|  | D1.3年降水量 | 钉螺依赖静水环境（如塘堰、沟渠），相关文献显示年降水量>800mm地区水体滞留时间长，适宜钉螺繁殖 | 统计气象站全年实测降水量总和（mm） |  |  |  |  |  |  |  |  |  |
|  | D1.4年平均相对湿度（删除！重要性、可操作性评分低） | 年平均相对湿度是指一年内所有小时测量得到的相对湿度值的平均数。反映了地区长期的湿度状况，对钉螺生存环境的长期适宜性具有影响 | 统计气象站全年8760h相对湿度实时监测值的平均值（%） |  |  |  |  |  |  |  |  |  |
|  | **D2 地形因素** | | | | | | | | | | | |
|  | D2.1 土壤类型 | 不同的土壤类型对钉螺的生存影响不同，适宜其生存的土壤通常为具有较好的保水能力黏土、壤土；沙质土、贫瘠土壤或排水性极强的土壤则相反 |  |  |  |  |  |  |  |  |  |  |
|  | D2.2 高程 | 血吸虫病流行区的海拔高度，影响了温度、湿度、洪水淹没情况，从而影响钉螺的生存与扩散 | 统计当地的海拔高度（m） |  |  |  |  |  |  |  |  |  |
|  | D2.3 植被覆盖率 | 血吸虫病流行区内植被的覆盖程度，影响土壤湿度和微环境，从而影响钉螺的栖息地稳定性。如高植被覆盖（湿地、灌丛），易形成适宜钉螺生境；而低植被覆盖（裸地、城镇），不适宜钉螺生存，风险较低 | 植被类型可参考全国血吸虫病监测方案（2025版）表6 钉螺监测调查表，植被种类：1.杂草，2.芦苇，3.树林，4.水稻，5.旱地作物，6.其它；并给出实际植被占比情况 |  |  |  |  |  |  |  |  |  |
|  | D2.4 土地利用方式 | 不同的土地利用类型，如农田、水体、湿地、建设用地等，对人畜疫水接触和血吸虫病传播具有重要影响 |  |  |  |  |  |  |  |  |  |  |
|  | **D3 自然灾害** | | | | | | | | | | | |
|  | D3.1 发生洪涝的次数 | 该地区发生的洪涝/内涝事件次数，包括江河湖泊水位异常上涨导致的大规模淹没、农田受灾等情况，反映该地区近年洪涝历史及其对钉螺栖息地的潜在影响 | 洪涝/内涝发生次数（次） |  |  |  |  |  |  |  |  |  |
|  | D3.2 发生干旱的次数 | 该地区因降水异常偏低（满足省级或国家级干旱预警标准）引发的持续性缺水事件发生次数，反应该地区干旱历史及其对钉螺栖息地稳定性的影响 | 干旱发生次数（次） |  |  |  |  |  |  |  |  |  |
|  | **D4生态防控工程实施情况** | | | | | | | | | | | |
|  | D4.1 水利工程血吸虫病防控专项占比 | 过去五年内，在区域水利工程总量中，采用防螺技术（如沟渠硬化、抬洲降滩、涵闸设沉螺池、中层取水等专项工程）的水利工程占比 | （五年内）采用防螺技术水利工程数/水利工程总数*100（%） |  |  |  |  |  |  |  |  |  |
|  | D4.2湿地保护面积年度变化比例 | 在一年内，血吸虫病流行区新增的湿地保护面积与年初已保护湿地总面积的比值，反映湿地保护工作的整体进展，体现湿地保护政策的实施效果。 | 年末修复湿地总面积-年初修复湿地总面积）/年初修复湿地总面积*100（%）新增湿地保护面积/初始湿地保护面积*100（%） |  |  |  |  |  |  |  |  |  |
| **E社会因素** | **E1经济** | | | | | | | | | | | |
|  | E1.1县级财政水平 | 县级政府的年度财政收入水平（一般预算收入），反映地方经济实力及公共卫生资源的可用性 | 统计流行县县级政府年度一般预算收入（亿元） |  |  |  |  |  |  |  |  |  |
|  | E1.2农村居民人均收入 | 农村居民在一定时期内（通常为一年）人均可支配收入，反映个体经济能力和生活水平 | 流行县县级农村居民可支配收入之和/常住人口总数（元/人） |  |  |  |  |  |  |  |  |  |
|  | **E2机构能力建设** | | | | | | | | | | | |
|  | E2.1血防专项经费投入 | 县级流行区血吸虫病专项防治资金占当年卫生总预算的比例（包括中央拨款、地方配套、社会捐赠） | 流行县血吸虫病专项防治资金/当年卫生总预算*100（%） |  |  |  |  |  |  |  |  |  |
|  | E2.2血防专职专业人员数 | 从事血吸虫病预防、诊断、治疗及健康教育的全职专业人员数量（含疾控、医院） | 统计流行县疾控、医院、全职专业人员总数(人) |  |  |  |  |  |  |  |  |  |
|  | E2.3血防实验室监测能力（年检测样品量） | 血吸虫病实验室每年完成的血清学、病原学检测样本总量 | 统计年检测样品量（件） |  |  |  |  |  |  |  |  |  |
|  | E2.4血防物资储备情况 | 血吸虫病防控物资（药品、防护用具、消杀器械）的实有库存量，按每月最低消耗量折算后，可支撑的应急响应时间（月份数） | Min（S_药品_/M_药品_，S_防护用具_/M_防护用具_，S_消杀器械_/M_消杀器械_）  S：实有库存；M：汛期月份最低消耗 |  |  |  |  |  |  |  |  |  |
|  | **E3水和卫生设施（WASH）** | | | | | | | | | | | |
|  | E3.1无害化卫生厕所覆盖率 | 无害化卫生厕所指采用沼气池、三格式、粪尿分流等工艺处理粪便的卫生厕所。其覆盖率是指达到无害化卫生厕所标准的户数占总农户数的比例 | 达无害化卫生厕所标准的户数/总农户数*100（%） |  |  |  |  |  |  |  |  |  |
|  | E3.2集中式供水安全覆盖率 | 拥有符合国家生活饮用水卫生标准（GB 5749-2022）的饮用水的居民人口比例，反映居民饮用水安全水平。 | 可获得符合 GB5749-2022 标准饮用水的人口 / 总人口 × 100%（%） |  |  |  |  |  |  |  |  |  |
|  | **E4精准防控能力** | | | | | | | | | | | |
|  | E4.1血吸虫病人规范性随访率 | 按照“行动方案”要求，通过电话、短信、登门、检查等规范化方式开展随访的血吸虫病病人数占登记在册的血吸虫病病人数 | 随访血吸虫病病人数/在册血吸虫病病人数*100（%） |  |  |  |  |  |  |  |  |  |
|  | E4.2健康教育 | 重点人群包括渔船民、农民、休闲垂钓人员、和重大工程外来施工人员等。通过调查重点人群的血防知识知晓率来衡量 | 回答正确题目总数/应回答题目总数*100（%） |  |  |  |  |  |  |  |  |  |
|  | E4.3无人机技术新发现钉螺孳生地数 | 通过无人机AI识别并人工复核确认的新增钉螺孳生地数量（不含历史已知区域） |  |  |  |  |  |  |  |  |  |  |
|  | E4.4血防智能哨卡违规拦截成功率 | 血防哨卡在自动识别违规行为（如未穿防护服、接触疫水、携带钉螺等）后，通过物理屏障、语音警告、AI识别联动等方式成功阻断目标的百分比 | 成功拦截次数/总违规事件*100（%） |  |  |  |  |  |  |  |  |  |
| 对三级指标的增删意见（若需要增加指标，请注明增加指标的名称及其定义）： | | | | | | | | | | | | |

**第二轮**

**表1 血吸虫病传播风险评估一级指标评分表**

| **一级指标名称** | **一级指标定义** | **计算公式（单位）** | **合理性** | **重要性** | **可操作性** | **判断依据（1-3）** | | | | **熟悉程度** | **对一级指标修改意见** |
| --- | --- | --- | --- | --- | --- | --- | --- | --- | --- | --- | --- |
|  |  |  | **（1-5）** | **（1-5）** | **（1-5）** | **实践经验** | **理论依据** | **同行了解** | **直**  **觉** | **（1-5）** | **（指标名称、释义、分类及计算公式等）** |
| A 生物因素 | 在血吸虫病传播风险评估中，“生物因素”指直接参与或影响血吸虫生活史循环、病原体（血吸虫）传播及宿主感染的核心生物类要素。其通过调控病原体的生存、繁殖、扩散及宿主易感性，构成血吸虫病传播的生物学基础，是评估传播风险的关键维度。 | = $\sum_{k=1}^{n=4} Ak^{'}权重$ |  |  |  |  |  |  |  |  |  |
| B 自然环境因素 | 影响血吸虫病传播的各类自然环境要素及相关干预工程的统称。涵盖了气候、地形、水文、自然灾害等自然条件的被动调节作用。该类因素通过影响钉螺孳生地和疫水暴露环境，在血吸虫病传播链中构成重要的物理和生态调控基础 | = $\sum_{k=1}^{n=3} Bk^{'}权重$ |  |  |  |  |  |  |  |  |  |
| C 社会因素 | 影响血吸虫病传播的社会要素与主动防控措施的集合体，通过调控经济资源配置、机构效能、卫生设施水平及精准干预手段，动态管理人群活动与疫水暴露风险，同时在防控体系薄弱环节识别潜在风险点，构成传播链中社会与人文维度的调控基础 | = $\sum_{k=1}^{n=5} Ck^{'}权重$ |  |  |  |  |  |  |  |  |  |
| 对一级指标的增删意见（若需要增加指标，请注明增加指标的名称及其定义）： | | | | | | | | | | | |

**表2 血吸虫病传播风险评估二级指标评分表**

| **一级指标名称** | **二级指标名称** | **二级指标定义** | **计算公式（单位）** | **合理性** | **重要性** | **可操作性** | **判断依据（1-3）** | | | | **熟悉程度** | **对二级指标修改意见** |
| --- | --- | --- | --- | --- | --- | --- | --- | --- | --- | --- | --- | --- |
|  |  |  |  | **（1-5）** | **（1-5）** | **（1-5）** | **实践经验** | **理论依据** | **同行了解** | **直**  **觉** | **（1-5）** | **（指标名称、释义、分类及计算公式等）** |
| A生物因素 | A1家畜传染源 | 家畜（如牛、羊）是血吸虫病的重要宿主，其通过粪便排出含血吸虫虫卵的排泄物污染环境（如水源、钉螺孳生地），直接驱动血吸虫生活史循环，是血吸虫病传播的核心生物传染源。 | = $\sum_{i=1}^{n=3} A1.i^{'}权重$ |  |  |  |  |  |  |  |  |  |
|  | A2野生动物传染源 | 野生动物（如野鼠、麋鹿等）是钉螺的自然宿主，其感染率反映生态传播风险 | = $\sum_{i=1}^{n=2} A2.i^{'}权重$ |  |  |  |  |  |  |  |  |  |
|  | A3 螺情 | 钉螺是血吸虫病的唯一中间宿主，其分布密度和感染状态直接决定尾蚴释放量。通过监测钉螺环境，评估尾蚴来源风险 | = $\sum_{i=1}^{n=5} A3.i^{'}权重$ |  |  |  |  |  |  |  |  |  |
|  | A4重点易感人群 | 根据《全国血吸虫病监测方案》以及《加快实现消除血吸虫病目标行动方案》，将易感人群按职业、行为或流动性特征聚焦于农民/渔船民，休闲垂钓人，重大工程外来施工人员以及抢险救灾官兵 | = $\sum_{i=1}^{n=4} A4.i^{'}权重$ |  |  |  |  |  |  |  |  |  |
| B 自然环境因素 | B1气候因素 | 通过温度、降水等气象条件直接影响钉螺生命周期与疫水暴露风险的环境变量 | = $\sum_{i=1}^{n=3} B1.i^{'}权重$ |  |  |  |  |  |  |  |  |  |
|  | B2地形因素 | 通过土壤、高程、植被等地理特征间接调控钉螺分布，决定传播路径的易感性 | = $\sum_{i=1}^{n=4} B2.i^{'}权重$ |  |  |  |  |  |  |  |  |  |
|  | B3水文因素 | 行政区域内与水体动态分布、流动规律及极端水文事件相关的自然特征，其通过调控钉螺孳生环境的稳定性、血吸虫尾蚴扩散等，直接或间接影响血吸虫病的传播风险 | = $\sum_{i=1}^{n=3} B3.i^{'}权重$ |  |  |  |  |  |  |  |  |  |
| C 社会因素 | C1 经济 | 通过县级财政水平与农村居民人均收入两个维度，评估血吸虫病防控工作中经济资源的可及性与个体行为的经济驱动能力，为防螺工程、健康教育及医疗保障等防控措施提供经济支撑 | = $\sum_{i=1}^{n=2} C1.i^{'}权重$ |  |  |  |  |  |  |  |  |  |
|  | C2机构能力建设 | 通过疾控机构网络覆盖、专业人员配置及应急响应效能，保障防控措施的执行力度 | = $\sum_{i=1}^{n=6} C2.i^{'}权重$ |  |  |  |  |  |  |  |  |  |
|  | C3水和卫生设施 | 安全饮水、普及卫生厕所是减少疫水接触风险，阻断传播途径的有效措施 | = $\sum_{i=1}^{n=2} C3.i^{'}权重$ |  |  |  |  |  |  |  |  |  |
|  | C4生态防控工程实施情况 | 通过水利工程、湿地保护等生态工程措施，主动调控钉螺孳生环境，阻断血吸虫病传播链的实施成效评估 | = $\sum_{i=1}^{n=2} C4.i^{'}权重$ |  |  |  |  |  |  |  |  |  |
|  | C5精准防控能力 | 通过科学监测任务的高效完成、重点人群健康教育的广泛覆盖，以及血防智能哨卡对违规行为的有效拦截，针对血吸虫病传播链关键节点实施分级管理和精准阻断，提升防控措施的针对性和实效性 | = $\sum_{i=1}^{n=3} C5.i^{'}权重$ |  |  |  |  |  |  |  |  |  |
| 对二级指标的增删意见（若需要增加指标，请注明增加指标的名称及其定义）： | | | | | | | | | | | | |

**表3 血吸虫病传播风险评估三级指标评分表**

| **一级指标名称** | **二三级指标名称** | **三级指标定义** | **计算公式（单位）** | **合理性** | **重要性** | **可操作性** | **判断依据（1-3）** | | | | **熟悉程度** | **对三级指标修改意见** |
| --- | --- | --- | --- | --- | --- | --- | --- | --- | --- | --- | --- | --- |
|  |  |  |  | **（1-5）** | **（1-5）** | **（1-5）** | **实践经验** | **理论依据** | **同行了解** | **直**  **觉** | **（1-5）** | **（指标名称、释义、分类及计算公式等）** |
| **A生物因素** | **A1家畜传染源** | | | | | | | | | | | |
|  | A1.1本地家畜粪检覆盖率 | 在本年度内，县级疾病预防控制机构会同当地动物疫病防控机构以监测范围内的本地存栏家畜（牛、羊等）为对象，是否采用病原学（毛蚴孵化法）检测感染的比例 | 采用病原学查病本地家畜数/本地存栏家畜总数*100（%） |  |  |  |  |  |  |  |  |  |
|  | A1.2引入家畜粪检覆盖率 | 从血吸虫病流行区引进的家畜（牛、羊等）由于运输、检疫等因素影响，可能成为重要输入性传染源。依照监测方案，采用病原学（毛蚴孵化法）检测引进家畜感染情况的比例 | 采用病原学查病引入家畜数/引入家畜总数*100（%） |  |  |  |  |  |  |  |  |  |
|  | A1.3野粪阳性率 | 在人畜活动频繁的有螺环境中采集的哺乳动物（牛、羊、猪、马属、狗等）粪便样本中，检测到血吸虫虫卵的阳性比例，用以追踪血吸虫病传播链中野粪污染水源的风险 | 阳性野粪数/采集野粪总数*100（%） |  |  |  |  |  |  |  |  |  |
|  | **A2野生动物感染情况** | | | | | | | | | | | |
|  | A2.1野鼠感染率 | 野鼠监测是风险监测的重要一环，说明其在低流行态势下可能是重要的传染源之一。对人、畜可及的有螺环境或可疑钉螺孳生环境捕捉野鼠，并通过实验室检测了解该年度野鼠血吸虫感染情况 | 检测出阳性的野鼠只数/检测的野鼠总只数*100% |  |  |  |  |  |  |  |  |  |
|  | A2.2其它野生动物感染情况 | 由于生态保护、恢复计划的实施，野生动物在优化的生态环境中活动，可能成为血吸虫病的潜在传染源。在野鼠监测过程中若有发现野生动物的感染情况，可以及时发现并评估其对血吸虫病传播的风险 | 是否发现有感染血吸虫病的野生动物（是=1/否=0） |  |  |  |  |  |  |  |  |  |
|  | **A3螺情** | | | | | | | | | | | |
|  | A3.1查出现有钉螺面积比例 | 在本年度内，县级行政区域内实际存在钉螺的孳生面积占调查范围内总查螺面积的比例。其通过量化钉螺分布的密集程度，直接反映区域内钉螺种群存续状态及血吸虫传播链的活跃性。 | 本年度内查出有螺总面积/同期查螺总面积*100% |  |  |  |  |  |  |  |  |  |
|  | A3.2新发现钉螺面积 | 根据监测方案对监测范围内螺情调查时，发现历史上从未出现钉螺的环境中，首次查到有钉螺分布的钉螺孳生环境面积 | 统计本年度首次查到有钉螺分布的孳生环境面积（m^2^） |  |  |  |  |  |  |  |  |  |
|  | A3.3复现钉螺面积 | 在历史有螺环境内，经防治曾被确认消灭，但连续两年及以上再度查到有活螺分布的钉螺孳生环境面积 | 统计本年度复现的钉螺孳生环境面积（m^2^） |  |  |  |  |  |  |  |  |  |
|  | A3.4活螺平均密度 | 根据监测方案，每年度在监测范围内调查获得每单位面积（通常为0.1平方米）中活钉螺的数量 | 捕获活螺数/调查框数（0.1/m^2^） |  |  |  |  |  |  |  |  |  |
|  | A3.5钉螺核酸阳性环境数 | 在本年度年度，县级行政区域内开展监测点监测和风险监测中，从不同自然环境（如沟渠、滩地、池塘等钉螺孳生地）中采集钉螺样本，通过核酸检测（如LAMP等分子生物学技术）确认有核酸阳性的环境数量 | 监测发现的钉螺核酸阳性环境数量（个） |  |  |  |  |  |  |  |  |  |
|  | **A4重点易感人群** | | | | | | | | | | | |
|  | A4.1高暴露职业人群（农民/渔船民）比例 | 在血吸虫病流行区域内，从事水稻种植、水产养殖、船舶驾驶等高暴露职业的常住及流动人口占总人口的比例 | （农民人数+渔船民人数）/总人口*100（%） |  |  |  |  |  |  |  |  |  |
|  | A4.2休闲垂钓人员数 | 在疫区水体（如湖泊、池塘、沟渠等）进行垂钓的人员数量，按人次统计（同一人多次垂钓计多次） | 统计垂钓的人员数量(人次/月) |  |  |  |  |  |  |  |  |  |
|  | A4.3重大工程外来施工人员数 | 在血吸虫病流行区内，因基建、水利、能源等重大项目需短期集中居住（≥3个月）的外地务工人员总数 | 各项目合同外地务工人员总数（人） |  |  |  |  |  |  |  |  |  |
|  | A4.4抢险救灾官兵人员数 | 因参与抢险救灾、应急救援等任务（如抗洪、抗震、疫情防控等）而短期居住（≤30天）于血吸虫病流行区的现役官兵、武警、消防救援人员等群体中，任务期间实际进入钉螺分布区或接触疫水环境（如河滩、沟渠）的人数。 | 抢险救灾的官兵总数（人） |  |  |  |  |  |  |  |  |  |
| **B 自然环境因素** | **B1 气候因素** | | | | | | | | | | | |
|  | B1.1 年平均气温 | 年平均气温决定区域是否具备钉螺长期存活条件，且血吸虫尾蚴在水中存活、释放均受温度影响 | 统计全年气温的平均值（℃） |  |  |  |  |  |  |  |  |  |
|  | B1.2 一月平均最低气温 | 熟一月通常是冬季最冷月份，低温会使钉螺进入休眠，而极端低温可直接杀死钉螺成体及虫卵 | 统计一月份每日最低气温的平均值（℃） |  |  |  |  |  |  |  |  |  |
|  | B1.3年降水量 | 钉螺依赖静水环境（如塘堰、沟渠），相关文献显示年降水量>800mm地区水体滞留时间长，适宜钉螺繁殖 | 统计气象站全年实测降水量总和（mm） |  |  |  |  |  |  |  |  |  |
|  | **B2 地形因素** | | | | | | | | | | | |
|  | B2.1 高程 | 血吸虫病流行区的海拔高度，影响了温度、湿度、洪水淹没情况，从而影响钉螺的生存与扩散 | 统计当地的海拔高度（m） |  |  |  |  |  |  |  |  |  |
|  | B2.2 植被覆盖率 | 血吸虫病流行区内植被的覆盖程度，影响土壤湿度和微环境，从而影响钉螺的栖息地稳定性。如高植被覆盖（湿地、灌丛），易形成适宜钉螺生境；而低植被覆盖（裸地、城镇），不适宜钉螺生存，风险较低 | 植被类型可参考全国血吸虫病监测方案（2025版）表6 钉螺监测调查表，植被种类：1.杂草，2.芦苇，3.树林，4.水稻，5.旱地作物，6.其它；并给出实际植被占比情况 |  |  |  |  |  |  |  |  |  |
|  | B2.3 土壤湿度 | 血吸虫病流行区内，表层土壤（0-20cm）的水分含量水平，通过直接调控土壤湿润度，作用于钉螺的生存、繁殖及活动能力。高湿度环境为钉螺提供了适宜的孳生条件；低湿度环境则因土壤水分匮乏，限制钉螺存活 | 统计当地的NDWI_soil（土壤湿度） |  |  |  |  |  |  |  |  |  |
|  | B2.4农田比例 | 农田（尤其水田、灌溉旱田）因长期或季节性积水、土壤湿润，常成为钉螺的适宜孳生地。在血吸虫病流行区内，农田面积占县域行政区域总面积的比例，是反映区域土地利用类型中农业生产用地占比的关键指标。其通过直接关联钉螺孳生环境的稳定性和人类生产活动强度，间接调控血吸虫传播风险 | 农田面积/县域行政区域总面积*100（%） |  |  |  |  |  |  |  |  |  |
|  | **B3 水文因素** | | | | | | | | | | | |
|  | B3.1 水系密度 | 在血吸虫病流行区内，县域行政区域内自然与人工水体的发育程度及空间分布密集度，是综合反映区域水网发达程度。水系发达地区湿润、钉螺生存环境优越。 | （县域内河流总长度 + 沟渠总长度 + 其他线性水体周长） / 县域行政区域总面积*100（%）（km/km^2^） |  |  |  |  |  |  |  |  |  |
|  | B3.2 发生洪涝的次数 | 该地区发生的洪涝事件次数，包括江河湖泊水位异常上涨导致的大规模淹没、农田受灾等情况，反映该地区近年洪涝历史及其对钉螺栖息地的潜在影响 | 洪涝发生次数（次） |  |  |  |  |  |  |  |  |  |
|  | B3.3 发生干旱的次数 | 该地区因降水异常偏低（满足省级或国家级干旱预警标准）引发的持续性缺水事件发生次数，反应该地区干旱历史及其对钉螺栖息地稳定性的影响 | 干旱发生次数（次） |  |  |  |  |  |  |  |  |  |
| **C社会因素** | **C1经济** | | | | | | | | | | | |
|  | C1.1县级财政水平 | 县级政府的年度财政收入水平（一般预算收入），反映地方经济实力及公共卫生资源的可用性 | 统计流行县县级政府年度一般预算收入（亿元） |  |  |  |  |  |  |  |  |  |
|  | C1.2农村居民人均收入 | 农村居民在一定时期内（通常为一年）人均可支配收入，反映个体经济能力和生活水平 | 流行县县级农村居民可支配收入之和/常住人口总数（元/人） |  |  |  |  |  |  |  |  |  |
|  | **C2机构能力建设** | | | | | | | | | | | |
|  | C2.1血防专项经费投入 | 县级流行区血吸虫病专项防治资金占当年卫生总预算的比例（包括中央拨款、地方配套、社会捐赠） | 流行县血吸虫病专项防治资金/当年卫生总预算*100（%） |  |  |  |  |  |  |  |  |  |
|  | C2.2血防专职人员数 | 从事血吸虫病预防、诊断、治疗及健康教育的全职专业人员数量（含疾控、医院） | 统计流行县疾控、医院全职专业人员总数(人) |  |  |  |  |  |  |  |  |  |
|  | C2.3血防兼职人员数 | 从事血吸虫病预防、诊断、治疗及健康教育的兼职人员数量（含疾控、医院、村医） | 统计流行县疾控、医院、村医兼职人员总数(人) |  |  |  |  |  |  |  |  |  |
|  | C2.4近三年血防实验室外部质量评价（EQA）达标年度比例 | 县级血防实验室近三年内（2022-2024）参加省级或国家血吸虫病实验室外部质量评价（EQA，实验室间比对），且结果合格率 ≥ 90% 的年度在总评估年度中的比例。该指标反映血防实验室检测能力的稳定性与持续质量水平。 | N达标年度/ N评估年度​*100（%） （N达标年度​ = 近三年中 EQA 合格率 ≥ 90% 的年度数；N评估年度​ = 近三年参加 EQA 的年度总数） |  |  |  |  |  |  |  |  |  |
|  | C2.5年专业培训次数 | 在本年度内，机构组织或参与的与血吸虫病防控、监测、实验室检测等相关的专业培训次数，包括内部培训、外部培训、线上培训与线下培训，需有培训记录或证明。 | 年度内开展的相关培训活动次数总和（次） |  |  |  |  |  |  |  |  |  |
|  | C2.6汛期月份血防物资储备情况 | 血吸虫病防控物资（药品、防护用具、消杀器械）的实有库存量，按汛期月份最低消耗量折算后，可支撑的应急响应时间（月份数） | Min（S_药品_/M_药品_，S_防护用具_/M_防护用具_，S_消杀器械_/M_消杀器械_）  S：实有库存；M：汛期月份最低消耗 |  |  |  |  |  |  |  |  |  |
|  | **C3水和卫生设施** | | | | | | | | | | | |
|  | C3.1无害化卫生厕所覆盖率 | 无害化卫生厕所指采用沼气池、三格式、粪尿分流等工艺处理粪便的卫生厕所。其覆盖率是指达到无害化卫生厕所标准的户数占总农户数的比例 | 达无害化卫生厕所标准的户数/总农户数*100（%） |  |  |  |  |  |  |  |  |  |
|  | C3.2集中式供水安全覆盖率 | 拥有符合国家生活饮用水卫生标准（GB 5749-2022）的饮用水的居民人口比例，反映居民饮用水安全水平。 | 可获得符合 GB5749-2022 标准饮用水的人口 / 总人口 × 100%（%） |  |  |  |  |  |  |  |  |  |
|  | **C4生态防控工程实施情况** | | | | | | | | | | | |
|  | C4.1水利工程血吸虫病防控专项占比 | 过去五年内，在区域水利工程总量中，采用防螺技术（如沟渠硬化、抬洲降滩、涵闸设沉螺池、中层取水等专项工程）的水利工程占比 | （五年内）采用防螺技术水利工程数/水利工程总数*100（%） |  |  |  |  |  |  |  |  |  |
|  | C4.2湿地保护面积增加比例 | 在本年度内，县级行政区域内通过自然保护地建设、生态修复等保护措施，实际新增的湿地保护面积占年初湿地保护面积的比例。通过量化湿地保护与修复的成效，间接反映区域钉螺孳生环境的调控能力 | 新增湿地保护面积/年初湿地保护面积*100（%） |  |  |  |  |  |  |  |  |  |
|  | **C5精准防控能力** | | | | | | | | | | | |
|  | C5.1监测任务完成率 | 本年度内，县级行政区域内计划开展的各类血吸虫病监测任务（包括环境钉螺监测、感染者检测、家畜检测等）实际完成数量占计划任务总量的比例。其通过量化监测工作的执行力度与规范性，直接反映监测体系的运行效率和风险发现能力 | 随访血吸虫病病人数/在册血吸虫病病人数*100（%） |  |  |  |  |  |  |  |  |  |
|  | C5.2健康教育 | 重点人群包括渔船民、农民、休闲垂钓人员、和重大工程外来施工人员等。通过调查重点人群的血防知识知晓率来衡量 | 回答正确题目总数/应回答题目总数*100（%） |  |  |  |  |  |  |  |  |  |
|  | C5.3是否建立智能哨卡 | 指县域内是否已建设并投入使用基于信息化、智能化技术的血吸虫病防控哨卡系统。该系统依托大数据、物联网、视频监控与智能识别等手段，对跨区域流动人员、牲畜及可能的传染源进行监测和预警，旨在阻断血吸虫病传播链条中的高风险环节。 | 是否建立血防智能哨卡（是=1/否=0） |  |  |  |  |  |  |  |  |  |
| 对三级指标的增删意见（若需要增加指标，请注明增加指标的名称及其定义）： | | | | | | | | | | | | |

**专家基本情况表**

*注：选择题请直接点击方框进行选择，填空题请在横线上填写内容

1. 您的姓名：________________
2. 您的性别：________
3. 您的年龄：________岁
4. 您的工作年限：________年
5. 您的最高学历是（单选）

本科 硕士 博士

1. 您的专业职称是（单选）

正高级 副高级

1. 您现在的工作单位（单选）

高等医学院校 卫生行政部门 医院 疾病预防控制中心

1. 您的联系方式 (电话或常用邮箱) ：________________
