# Supplementary material for: A county-level indicator framework for assessing schistosomiasis transmission risk in post-transmission-interruption China
Source: Infect Dis Poverty. 2026 May 18;15:58. doi: 10.1186/s40249-026-01453-6 (PMC13182038; doi:10.1186/s40249-026-01453-6)
Supplement: Supplementary file 2 — Additional file 2: Supplementary Table 1. Operational definitions, calculation methods, units, and directionality of the final third-level indicators in the county-level schistosomiasis transmission risk assessment framework [file 40249_2026_1453_MOESM2_ESM.docx]

| ***Supplementary Table 1. Operational definitions, calculation methods, units, and directionality of the final third-level indicators in the county-level schistosomiasis transmission risk assessment framework*** | | | | | |
| --- | --- | --- | --- | --- | --- |
| ***Code*** | ***Third-level indicator*** | ***Operational description*** | ***Formula/ Calculation*** | ***Unit*** | ***Directionality*** |
| A1.1 | Coverage of fecal examination in local livestock | Proportion of locally raised livestock (e.g., cattle and sheep/goats) within the surveillance area that underwent **etiological examination by miracidial hatching test** for schistosome infection during the reporting year. | Number of local livestock examined by miracidial hatching test / total number of local livestock in stock (cattle + sheep/goats) × 100% | % | Risk-negative |
| A1.2 | Coverage of fecal examination in introduced livestock | Proportion of livestock introduced from schistosomiasis-endemic areas that underwent **etiological examination by miracidial hatching test** for schistosome infection during the reporting year. | Number of introduced livestock examined by miracidial hatching test / total number of introduced livestock (cattle + sheep/goats) × 100% | % | Risk-negative |
| A1.3 | Positivity rate of wild feces samples | Proportion of fecal samples collected from free-ranging mammals in snail-infested environments with frequent human or animal activity that tested positive for schistosome eggs; pen-collected feces were excluded. | Number of wild feces positive for schistosome eggs / total number of wild fecal samples collected × 100% | % | Risk-positive |
| A2.1 | Infection rate in wild rodents | Wild rodent surveillance is an important component of risk monitoring, as rodents may serve as a potential infection source under low-endemic conditions. Rodents were trapped in snail-infested environments or suspected snail habitats accessible to humans or livestock, and schistosome infection was determined by laboratory examination. | Number of wild rodents positive for schistosome infection / total number of wild rodents examined × 100% | % | Risk-positive |
| A2.2 | Infection status of other wild animals | With ecological protection and habitat restoration, wild animals may act as potential sources of schistosome infection. Detection of infected wild animals during rodent surveillance helps identify and assess their potential role in transmission risk. | Presence of schistosome-infected wild animals detected (yes = 1; no = 0) | (0/1) | Risk-positive |
| A3.1 | Detection rate of existing snail habitats | Proportion of the surveyed snail-search area in which living Oncomelania snails were detected during the reporting year, reflecting the persistence and distribution intensity of snail habitats. | Total area with snails detected in the reporting year / total area surveyed for snails in the same year × 100% | % | Risk-positive |
| A3.2 | Area of newly detected snail habitats | Area of snail habitats where Oncomelania snails were detected for the first time in environments with no previous history of snail occurrence during the reporting year. | Total area of habitats newly found to contain snails in the reporting year | m² | Risk-positive |
| A3.3 | Area of reemerged snail habitats | Area of historical snail habitats where snails had previously been declared eliminated but were detected again after at least two consecutive years of absence during the reporting year. | Total area of re-emergent snail habitats detected in the reporting year | m² | Risk-positive |
| A3.4 | Mean density of live snails | Annual average number of living Oncomelania snails detected per survey frame within the surveillance area, based on routine snail surveys. | Number of living snails captured / number of survey frames examined | snails/0.1 m² frame | Risk-positive |
| A3.5 | Nucleic acid-positive snail habitats | Number of environmental sites where snail samples tested positive by nucleic acid detection methods (e.g., LAMP) during routine or risk-based surveillance in the reporting year. | Number of environments with positive snail nucleic acid detection | No. | Risk-positive |
| A4.1 | Water-contact exposure rate | Proportion of individuals with positive schistosomiasis questionnaire screening results among occupational groups with frequent water contact (e.g., rice farmers and aquaculture workers), reflecting the contribution of high-exposure groups to local transmission risk. | Number of individuals positive by schistosomiasis questionnaire screening / total number of individuals screened by questionnaire × 100% | % | Risk-positive |
| A4.2 | Recreational fishers | Number of people engaging in recreational fishing in endemic water bodies (e.g., lakes, ponds, and ditches), counted by person-times; repeated fishing by the same person was counted repeatedly. | Total number of recreational fishing person-times | person-times/month | Risk-positive |
| A4.3 | Migrant construction workers | Total number of non-local workers temporarily residing for major infrastructure, water conservancy, energy, or other large projects in schistosomiasis-endemic areas for at least 3 months. | Total number of non-local workers recorded in project contracts | persons | Risk-positive |
| A4.4 | Military personnel deployed for flood relief | Number of military, armed police, firefighting, or other emergency rescue personnel temporarily stationed in schistosomiasis-endemic areas for rescue or disaster-relief tasks who actually entered snail-infested areas or contacted potentially infested water during the mission period. | Total number of rescue personnel entering snail-infested areas or contacting risky water environments | persons | Risk-positive |
| B1.1 | Annual mean temperature | Annual mean temperature determines whether local conditions are suitable for long-term survival of Oncomelania snails, and also affects the survival and release of schistosome cercariae in water. | Mean value of air temperature over the reporting year | °C | Risk-positive |
| B1.2 | Mean minimum temperature in January | January is usually the coldest month of the year. Low temperature can induce snail dormancy, while extreme cold may directly kill adult snails and schistosome eggs. | Mean value of daily minimum temperature in January | °C | Risk-positive |
| B1.3 | Annual precipitation | *Oncomelania* snails depend on relatively stable water environments such as ponds and ditches. Areas with annual precipitation above 800 mm tend to have longer water retention, which is favorable for snail breeding. | Total recorded precipitation over the reporting year | mm | Risk-positive |
| B2.1 | Elevation | Elevation of schistosomiasis-endemic areas, which influences temperature, humidity, and flooding patterns and thereby affects the survival and spread of *Oncomelania* snails. | Mean elevation of endemic areas | m | Risk-negative |
| B2.2 | Vegetation coverage | Degree of vegetation cover in schistosomiasis-endemic areas, which affects soil moisture and microenvironmental conditions and thereby influences habitat suitability and stability for *Oncomelania* snails. Vegetation types may be classified according to the National Schistosomiasis Surveillance Scheme (2025), including weeds, reeds, woodland, rice, dryland crops, and others. | Proportion of actual vegetation cover by type in endemic areas | % | Risk-positive |
| B2.3 | Soil moisture | Moisture level of topsoil (0–20 cm) in schistosomiasis-endemic areas, which directly affects snail survival, reproduction, and activity. Higher soil moisture favors suitable snail habitats, whereas lower soil moisture limits snail survival. | NDWI_soil value for local soil moisture | Index | Risk-positive |
| B2.4 | Farmland proportion | Proportion of farmland area within the county administrative area. Farmland, especially paddy fields and irrigated dryland, often provides suitable habitats for *Oncomelania* snails because of persistent or seasonal soil moisture and waterlogging. | Farmland area / total county administrative area × 100% | % | Risk-positive |
| B3.1 | Density of snail-infested water systems | Ratio of the total length of ditches with snail distribution to the total county administrative area, reflecting the development and spatial density of snail-infested water bodies in the area. Higher density usually indicates a more developed water network and wetter environments suitable for snail survival. | Total length of snail-infested ditches / total county administrative area | km/km² | Risk-positive |
| B3.2 | Flood events | Number of flood events in the area, including large-scale inundation caused by abnormal rises in river, lake, or reservoir water levels, reflecting the recent flood history and its potential impact on snail habitats. | Number of flood events | times | Risk-positive |
| B3.3 | Drought events | Number of persistent water-shortage events caused by abnormally low precipitation and meeting provincial- or national-level drought warning criteria, reflecting the recent drought history and its impact on the stability of snail habitats. | Number of drought events | times | Risk-negative |
| C1.1 | County fiscal capacity | Annual general budget revenue of the county government, reflecting local economic capacity and the availability of public health resources. | Annual general budget revenue of the county government in endemic counties | 100 million CNY | Risk-negative |
| C1.2 | Per capita income of rural residents | Per capita disposable income of rural residents within a given period (usually one year), reflecting individual economic capacity and living standards. | Annual per capita disposable income of rural residents in endemic counties | CNY/person | Risk-negative |
| C2.1 | Investment in schistosomiasis control | Proportion of county-level dedicated funding for schistosomiasis control in the total annual health budget, including central transfers, local matching funds, and social donations. | Dedicated schistosomiasis control funding in endemic counties / total annual health budget × 100% | % | Risk-negative |
| C2.2 | Full-time schistosomiasis control staff | Number of full-time personnel engaged in schistosomiasis prevention, diagnosis, treatment, and health education, including staff from CDCs and hospitals. | Total number of full-time professional staff in county CDCs and hospitals | persons | Risk-negative |
| C2.3 | Part-time schistosomiasis control staff | Number of part-time personnel engaged in schistosomiasis prevention, diagnosis, treatment, and health education, including staff from CDCs, hospitals, and village clinics. | Total number of part-time staff in county CDCs, hospitals, and village clinics | persons | Risk-negative |
| C2.4 | EQA compliance rate | Whether the county schistosomiasis laboratory participated in provincial- or national-level external quality assessment (EQA) during the reporting year and achieved a qualification rate of at least 90%, reflecting the stability and quality of laboratory testing capacity. | Whether the EQA qualification rate was ≥ 90% (yes = 1; no = 0) | Binary (0/1) | Risk-negative |
| C2.5 | Annual professional training sessions | Number of professional trainings related to schistosomiasis control, surveillance, or laboratory testing organized by national-, provincial-, or municipal-level health authorities or professional institutions for county-level agencies during the reporting year. | Total number of relevant training activities received during the reporting year | times | Risk-negative |
| C2.6 | Flood-season control material stockpile | Number of months that key schistosomiasis control supplies available at the beginning of the flood season (May–October) could support emergency response, based on the minimum support time among chemotherapy drugs, protective equipment, and molluscicides. | (T_i = S_i / M_i); (S_i =) previous-year carryover + procurement at the beginning of the year − consumption from January to April; (M_i =) highest monthly consumption during May–October; final indicator (T = \min(T_{\text{chemotherapy drugs}}, T_{\text{protective equipment}}, T_{\text{molluscicides}})) | months | Risk-negative |
| C3.1 | Sanitary toilet coverage | Proportion of rural households with toilets meeting the standard for harmless sanitary toilets, including systems using biogas digesters, three-compartment septic tanks, or feces–urine separation for safe fecal treatment. | Number of households meeting the standard for harmless sanitary toilets / total number of rural households × 100% | % | Risk-negative |
| C3.2 | Safe water supply coverage | Proportion of the population with access to drinking water meeting the national Standards for Drinking Water Quality (GB 5749-2022), reflecting the level of drinking water safety among residents. | Population with access to drinking water meeting GB 5749-2022 / total population × 100% | % | Risk-negative |
| C4.1 | Coverage of SRRP* in Yangtze-connected waterways | Proportion of key snail-infested river-connected channels within the county that have been treated with schistosomiasis control engineering measures, reflecting the extent and coverage of water conservancy-based schistosomiasis control. | Number of key snail-infested river-connected channels treated / total number of key snail-infested river-connected channels × 100% | % | Risk-negative |
| C4.2 | Annual growth rate of protected wetland area | Proportion of newly added protected wetland area during the reporting year relative to the protected wetland area at the beginning of the year, reflecting the effectiveness of wetland protection and ecological restoration and, indirectly, the capacity to regulate snail habitats. | Newly added protected wetland area / protected wetland area at the beginning of the year × 100% | % | Risk-positive |
| C5.1 | Mobile-population surveillance task completion rate | Proportion of the planned annual surveillance target for mobile populations aged ≥6 years that was actually completed by county-level agencies in schistosomiasis-endemic areas. Priority groups included migrants engaged in crop planting, aquaculture, fishing and water transport, flood control and emergency rescue, and construction work. | Number of mobile population individuals actually surveyed / number of individuals planned for annual surveillance × 100% | % | Risk-negative |
| C5.2 | Health education coverage | Assessed by the awareness rate of schistosomiasis prevention and control knowledge among key populations. | Number of correctly answered questions / total number of questions to be answered × 100% | % | Risk-negative |
| C5.3 | Presence of smart sentinel sites | Whether an information-based and intelligent schistosomiasis control sentinel post system had been established and put into use within the county for monitoring and early warning of cross-regional movement of people, livestock, and other potential infection sources. | Whether an intelligent schistosomiasis control sentinel post had been established (yes = 1; no = 0) | Binary (0/1) | Risk-negative |

**Notes:** For **C5.1**, completion of the surveillance task was coded as 1, whereas not conducted or not completed was coded as 0. For **C5.2**, scores were assigned as follows: > 0.98 = 3, 0.95–0.98 = 2, 0.90–0.95 = 1, and not conducted = 0. **CNY** refers to Chinese yuan. **SRRP** refers to snail-related river regulation projects.
