# Supplementary material for: A county-level indicator framework for assessing schistosomiasis transmission risk in post-transmission-interruption China
Source: Infect Dis Poverty. 2026 May 18;15:58. doi: 10.1186/s40249-026-01453-6 (PMC13182038; doi:10.1186/s40249-026-01453-6)
Supplement: Supplementary file 5 — Additional file 5: Supplementary Table 2. Two rounds of Delphi consultation and indicator refinement [file 40249_2026_1453_MOESM5_ESM.docx]

| ***Supplementary Table 2. Two rounds of Delphi consultation and indicator refinement*** | | | |
| --- | --- | --- | --- |
| ***Round 1 Delphi consultation*** | ***modification status*** | ***Round 2 Delphi consultation*** | ***modification status*** |
| ***First-level indicators*** |  | ***First-level indicators*** |  |
| ***A.*** Source of infection | ***Merged*** | ***A.*** Biological factors | ***Retained*** |
| ***B.*** Route of transmission |  |  |  |
| ***C.*** Susceptible population |  |  |  |
| ***D.*** Environmental factors | ***Retained*** | ***B.*** Environmental factors | ***Retained*** |
| ***E.*** Social factors | ***Retained*** | ***C.*** Social factors | ***Retained*** |
| ***Second-level indicators*** |  | ***Second-level indicators*** |  |
| ***A1.*** Human infection status | ***Deleted*** |  |  |
| ***A2.*** Livestock infection status | ***Revised*** | ***A1.***Livestock sources of infection | ***Retained*** |
| ***A3.*** Wildlife infection status | ***Revised*** | ***A2.***Wildlife sources of infection | ***Retained*** |
| ***B1.*** Snail status | ***Retained*** | ***A3.***Snail status | ***Retained*** |
| ***B2.*** Water and environmental contamination status | ***Deleted*** |  |  |
| ***C1.*** Key populations | ***Retained*** | ***A4.*** Key populations | ***Retained*** |
| ***D1.*** Climatic factors | ***Retained*** | ***B1.*** Climatic factors | ***Retained*** |
| ***D2.*** Topographic factors | ***Retained*** | ***B2.*** Topographic factors | ***Retained*** |
| ***D3.*** Natural disasters | ***Revised*** | ***B3.*** Hydrographic factors | ***Retained*** |
| ***D4.*** Implementation of ecological control projects | ***Reassigned*** | ***C4.*** Implementation of ecological control projects | ***Retained*** |
| ***E1.*** Economic factors | ***Retained*** | ***C1.*** Economic factors | ***Retained*** |
| ***E2.*** Institutional capacity | ***Retained*** | ***C2.*** Institutional capacity | ***Retained*** |
| ***E3.*** WASH | ***Retained*** | ***C3.*** WASH | ***Retained*** |
| ***E4.*** Precision control | ***Revised*** | ***C5.*** Precision control capacity | ***Retained*** |
| ***Third-level indicators*** |  | ***Third-level indicators*** |  |
| ***A1.1.*** Seroprevalence among residents | ***Deleted*** |  |  |
| ***A1.2.*** Fecal prevalence among residents | ***Deleted*** |  |  |
| ***A1.3.*** Acute cases | ***Deleted*** |  |  |
| ***A1.4.*** Newly advanced cases | ***Deleted*** |  |  |
| ***A1.5.*** Newly chronic cases | ***Deleted*** |  |  |
| ***A1.6.*** Seroprevalence in mobile populations | ***Deleted*** |  |  |
| ***A1.7.*** Fecal Prevalence in mobile populations | ***Deleted*** |  |  |
| ***A1.8.*** Overall human infection prevalence | ***Deleted*** |  |  |
| ***A2.1.*** Infected local livestock | ***Revised*** | ***A1.1.*** Coverage of fecal examination in local livestock | ***Retained*** |
| ***A2.2.*** Infected introduced livestock | ***Revised*** | ***A1.2.*** Coverage of fecal examination in introduced livestock | ***Retained*** |
|  |  | ***A1.3.*** Positivity rate of wild feces samples | ***Retained*** |
| ***A3.1.*** Infection rate among wild rats | ***Retained*** | ***A2.1.*** Infection rate in wild rodents | ***Retained*** |
| ***A3.2.*** Infection status of other wild animals | ***Retained*** | ***A2.2.*** Infection status of other wild animals | ***Retained*** |
| ***B1.1.*** Area of snail habitat | ***Revised*** | ***A3.1.*** Detection rate of existing snail habitats | ***Retained*** |
| ***B1.2.*** Area of newly detected snail habitats | ***Retained*** | ***A3.2.***Area of newly detected snail habitats | ***Retained*** |
| ***B1.3.*** Area of reemerged snail habitats | ***Retained*** | ***A3.3.*** Area of reemerged snail habitats | ***Retained*** |
| ***B1.4.*** Mean density of live snails | ***Retained*** | ***A3.4.*** Mean density of live snails | ***Retained*** |
| ***B1.5.*** Density of infected snails | ***Merged and Revised*** | ***A3.5.*** Nucleic acid-positive snail habitats | ***Retained*** |
| ***B1.6.*** Infection Prevalence in Snails |  |  |  |
| ***B1.7.*** Frame occurrence rate of live snails | ***Deleted*** |  |  |
| ***B2.1.*** Positivity rate among sentinel mice | ***Deleted*** |  |  |
| ***B2.2.*** Positivity rate of field fecal samples | ***Reassigned*** |  |  |
| ***C1.1.*** Farmers and fishers | ***Retained*** | ***A4.1.*** Farmers and fishers | ***Revised*** |
| ***C1.2.*** Recreational fishers | ***Retained*** | ***A4.2.*** Recreational fishers | ***Retained*** |
| ***C1.3.*** Migrant construction workers | ***Retained*** | ***A4.3.*** Migrant construction workers | ***Retained*** |
|  | ***Added*** | ***A4.4.*** Military personnel deployed for flood relief | ***Retained*** |
| ***D1.1.*** Annual mean temperature | ***Retained*** | ***B1.1.*** Annual mean temperature | ***Retained*** |
| ***D1.2.*** Mean minimum temperature in January | ***Retained*** | ***B1.2.*** Mean minimum temperature in January | ***Retained*** |
| ***D1.3.*** Annual precipitation | ***Retained*** | ***B1.3.*** Annual precipitation | ***Retained*** |
| ***D1.4.*** Annual mean relative humidity | ***Deleted*** |  |  |
| ***D2.1.*** Elevation | ***Retained*** | ***B2.1.*** Elevation | ***Retained*** |
| ***D2.2.*** Vegetation coverage | ***Retained*** | ***B2.2.*** Vegetation coverage | ***Retained*** |
| ***D2.3.*** Soil type | ***Revised*** | ***B2.3.*** Soil moisture | ***Retained*** |
| ***D2.4.*** Land use type | ***Revised*** | ***B2.4.*** Farmland proportion | ***Retained*** |
| ***D3.1.*** Flood events | ***Added*** | ***B3.1.*** Density of water systems | ***Revised*** |
|  | ***Retained*** | ***B3.2.*** Flood events | ***Retained*** |
| ***D3.2.*** Drought events | ***Retained*** | ***B3.3.*** Drought events | ***Retained*** |
| ***D4.1.*** Schistosomiasis project coverage in water works | ***Reassigned*** | ***C4.1.*** Schistosomiasis project coverage in water works | ***Revised*** |
| ***D4.2.*** Annual change in wetland restoration area | ***Reassigned and Revised*** | ***C4.2.*** Annual growth rate of protected wetland area | ***Retained*** |
| ***E1.1.*** County fiscal capacity | ***Retained*** | ***C1.1.*** County fiscal capacity | ***Retained*** |
| ***E1.2.*** Per capita income of rural residents | ***Retained*** | ***C1.2.*** Per capita income of rural residents | ***Retained*** |
| ***E2.1.*** Investment in schistosomiasis control | ***Retained*** | ***C2.1.*** Investment in schistosomiasis control | ***Retained*** |
| ***E2.2.*** Schistosomiasis control staff | ***Added*** | ***C2.2.*** Full-time schistosomiasis control staff | ***Retained*** |
|  |  | ***C2.3.*** Part-time schistosomiasis control staff | ***Retained*** |
| ***E2.3.*** Annual laboratory testing capacity | ***Revised*** | ***C2.4.*** EQA* compliance rate | ***Retained*** |
| ***E2.4.*** Schistosomiasis control material stockpile | ***Added*** | ***C2.5.*** Annual professional training sessions | ***Retained*** |
|  | ***Revised*** | ***C2.6.*** Flood-season control material stockpile | ***Retained*** |
| ***E3.1.*** Sanitary toilet coverage | ***Retained*** | ***C3.1.*** Sanitary toilet coverage | ***Retained*** |
| ***E3.2.*** Safe water supply coverage | ***Retained*** | ***C3.2.*** Safe water supply coverage | ***Retained*** |
| ***E4.1.*** Patient follow-up coverage | ***Revised*** | ***C5.1.*** Surveillance task completion rate | ***Revised*** |
| ***E4.2.*** Health education coverage | ***Retained*** | ***C5.2.*** Health education coverage | ***Retained*** |
| ***E4.3.*** Drone-detected snail habitats | ***Deleted*** |  |  |
| ***E4.4.*** Violation detection rate by smart sentinel sites | ***Revised*** | ***C5.3.*** Presence of smart sentinel sites | ***Retained*** |

*EQA：External quality assessment.
